# Co-crystal polymorphism: five colourful forms of prothionamide-saccharin co-crystal

**DOI:** 10.1039/d6ra03965k

**Published:** 2026-08-25

**Authors:** Sonali Ghosh, Samina Easmin, Venkateswara Rao Pedireddi

**Affiliations:** a Solid State & Supramolecular Structural Chemistry Laboratory, Department of Chemistry, School of Basic Sciences, Indian Institute of Technology Bhubaneswar Argul Bhubaneswar 752 050 India sg46@iitbbs.ac.in +91 674 713 5378

## Abstract

Polymorphism in pharmaceutical co-crystals strongly influences solid-state properties and ultimately drug performance, yet the presence of multiple polymorphs within a co-crystal system remains uncommon, and it is very challenging to simultaneously isolate them. Herein, we report five distinct salt polymorphs (Forms I–V) of a 1 : 1 pharmaceutical co-crystal composed of an antituberculosis drug, prothionamide (PA), and the GRAS co-former saccharin (SA); they are obtained through solution-based crystallization by the controlled variation of the solvent and temperature. These forms show distinct morphologies (plate, block, hexagonal and rods) and vibrant colours (red, orange and yellow), as perceived by optical images. Single-crystal and powder X-ray diffraction analyses establish identical stoichiometry across all forms but reveal distinct unit cell parameters and packing motifs. Conformational analysis demonstrates that the inherent structural flexibility of a PA molecule plays a decisive role in enabling polymorphic diversity. Thermal characterization by differential scanning calorimetry (DSC) and variable-temperature powder X-ray diffraction (VTPXRD) shows that all the forms are stable and remain intact even under non-ambient conditions (for example, at high temperature), except Form III, which transforms into Form V upon heating. Furthermore, the stability of the polymorphs and the strength of their intermolecular interactions are evaluated by performing lattice energy and Hirshfeld surface analysis. Also, the distinct colours are substantiated with UV-visible and photoluminescence spectroscopic measurements, as well as computational studies.

## Abstract 2

Five colourful polymorphs (Forms I–V) of an antituberculosis drug, prothionamide, and saccharin were isolated using the slow evaporation method by altering the solvents under varied crystallization conditions.

## Graphical abstract

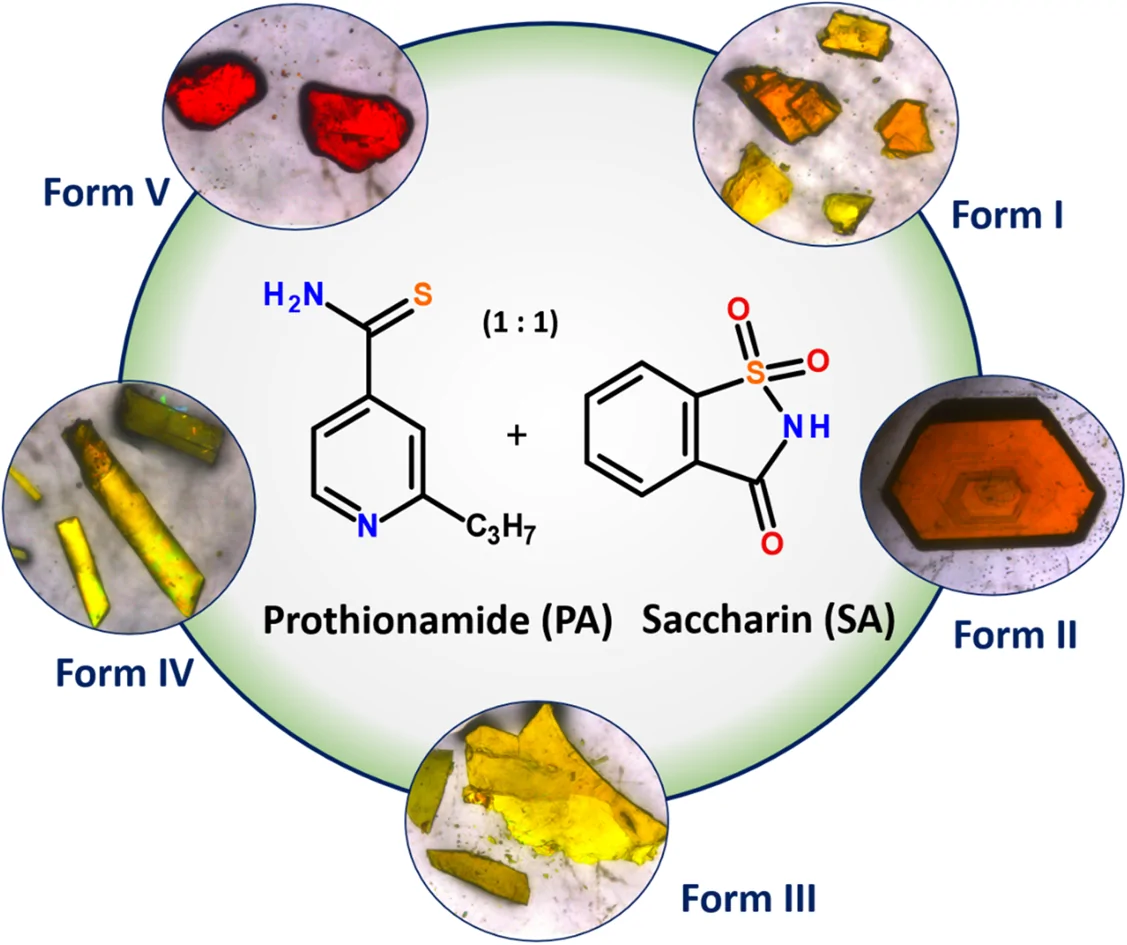

## Introduction

Within the realm of supramolecular chemistry, the ‘polymorphism’ phenomenon has been extensively studied in recent times, dealing with different crystals (structures) distinguishable by varied properties but formed by the same compound. Such different types of crystals are referred to as ‘polymorphs’.^1–6^ The phenomenon is more relevant in the contemporary arena of pharmaceutics with the evolution and evaluation of different polymorphs of various active pharmaceutical ingredients (APIs) that crystallize in different forms.^7^ In fact, the effective exploration and analysis of the polymorphic forms of APIs have emerged as a vital aspect in drug development and explorations because they significantly impact the pharmaceutical properties, like stability, solubility, bioavailability, and hygroscopicity.^8,9^ These properties directly influence drug manufacturing, formulation design, storage, distribution, administration, as well as therapeutic efficacy and safety, strongly highlighting the economic significance of novel polymorphs.^10–13^ The importance of polymorphism in pharmaceutics is well illustrated by the judicial battle over ranitidine polymorphs^14^ and is further reflected in the evolution of polymorphic forms for various versatile drugs, like paracetamol.^15–20^ However, the exploration of polymorphism in crystals is one of the challenging exercises in solid-state chemistry, though a few instances with numerous polymorphs are reported in the literature, like pyrazinamide (four),^21,22^ glycine (three),^23^ and ritonavir (four),^24^ particularly in the domain of pharmaceutically important molecules, APIs. However, one of the well-known compounds, ROY [5-methyl-2-[(2-nitrophenyl) amino]-3-thiophenecarbonitrile], is reported to form fourteen polymorphs to date.^25–29^

In general, polymorphism is considered to be a solvent-induced phenomenon as different polymorphs are generally obtained by carrying out the crystallization of a compound from different solvents/a mixture of solvents. However, the real driving force is the subtle intermolecular interactions that bind molecules together in the confined space (solid-state) and appear in different topologies, facilitated by the flexibility of the functional groups to orient into various conformations.^30–34^ In recent times, with the advancements in the characterization of solids with the highest possible precision, the polymorphs are prepared either directly or through the transformation of one form into another within the solid-state by subjecting the solids to external stimuli, like high temperature, pressure, and vacuum.^35,36^ Furthermore, the polymorphic forms are known to evolve with the seeding process.^37–40^

In crystal/structural chemistry, in recent times, studies pertaining to the development of multi-component crystals, referred to as co-crystals, have emerged as a research frontier primarily to create novel and fascinating archetypal structures with exotic applications in the domains that include organic synthesis (C–C bond formation), catalysis, and separation chemistry. In parallel, the co-crystals of pharmaceutically important molecules, like drugs, have shown profound significance, as the bio-properties of APIs, for example, solubility and bioavailability, can be well altered by co-crystallizing with Generally Recognized As Safe (GRAS) co-formers, authenticated by the US Food and Drug Administration (USFDA).

In contrast to the extensive research on single-component crystals, including pharmaceutically important molecules, APIs, the studies on polymorphic co-crystals are limited, with only a few studies known in the literature until recently. For instance, co-crystals like barbituric acid-urea,^41^ pimelic acid-4,4′-bipyridine,^42^ ethenzamide-gentisic acid,^43^ and furosemide-1,2-bis(4-pyridyl)ethylene^44^ exhibit a maximum of three polymorphs. Interestingly, co-crystals that exhibit more than three polymorphs are hitherto unknown.

In this regard, herein, we report, for the first time, a serendipitous discovery of an unusually high number of novel polymorphs (Five) of an antituberculosis drug (BCS class II), prothionamide (PA), with a GRAS compound, saccharin (SA). Similar to the well-known ‘ROY’ molecule, these forms exhibit distinct colours and morphologies, demonstrating that the structural flexibility of the thioamide (–CSNH_2_) and a substituted propyl group prevail in PA, suggesting a possible reason for the diverse polymorphic forms, as illustrated in the following sections.

## Experimental sections

### Materials and methods

All the chemicals and solvents were procured from Sigma-Aldrich at 99.9% purity (HPLC grade). Crystallization was carried out with these chemicals and solvents without further purification.

Here, five polymorphs were synthesized by taking an equimolar amount of both co-formers (PA and SA) and following the slow evaporation method under different crystallization conditions (like varied solvents and temperatures). One typical example was demonstrated by the formation of Form I polymorph.

A mixture of PA (0.1 mmol, 18.02 mg) with SA (0.1 mmol, 18.31 mg) was added to a 10 mL conical flask containing 5 mL of EtOAc solvent, and a homogeneous solution was prepared after gently heating in a water bath. The cooled solution was then kept at room temperature for crystallization by the slow evaporation method. Within 24 h, yellow-coloured block-shaped crystals appeared at the bottom of the conical flask.

#### Single-crystal X-ray diffraction (SCXRD) studies

The high-quality single crystals of all complexes were chosen using a microscope (Leica), and they were affixed to a glass fibre using a Paratone-*N* adhesive. Subsequently, the crystals were positioned on the goniometer of a Bruker single-crystal X-ray diffractometer (D8 VENTURE) equipped with a PHOTON 100 CMOS detector. The process of data collection was carried out smoothly without encountering any issues, and the crystals remained stable throughout. Data collection involved the utilization of Phi (*Φ*) and omega (Ω) scans. The structures were determined using an intrinsic phasing method, followed by refinement against *F*^2^ using the SHELXTL suite. Anisotropic methods were employed to refine the non-hydrogen atoms within the molecular complexes, while hydrogen atoms were either refined or positioned through calculations.^45^ The structural refinements resulted in the attainment of satisfactory *R*-factors, as detailed in Table 1. The computation of intermolecular interactions was performed using PLATON software^46^ (Table 2). For the creation of packing diagrams, Diamond (version 4.6.3)^47^ and Mercury (version 3.10.3)^48^ were employed.

**Table 1:** Crystallographic information for Forms I–V

Parameters	Form I	Form II	Form III	Form IV	Form V
Formula	C_16_H_17_N_3_O_3_S_2_	C_16_H_17_N_3_O_3_S_2_	C_16_H_17_N_3_O_3_S_2_	C_16_H_17_N_3_O_3_S_2_	C_16_H_17_N_3_O_3_S_2_
*M*_r_	363.32^a^	363.44	363.44	363.44	363.44
Crystal shape	Block	Hexagonal	Plate	Rod	Rectangular
Crystal size	0.28 × 0.24 × 0.21	0.14 × 0.12 × 0.10	0.29 × 0.26 × 0.22	0.24 × 0.12 × 0.09	0.25 × 0.20 × 0.12
Crystal color	Yellow	Orange	Yellow	Yellow	Red
Crystal system	Monoclinic	Triclinic	Triclinic	Monoclinic	Monoclinic
Space group	*P*2/*n*	*P*1̄	*P*1̄	*C*2/*c*	*P*2_1_/*c*
*T*, K	295 (2)	100 (2)	295 (2)	120 (2)	293 (2)
*λ*(Mo-K_α_)/Å	0.71073	0.71073	0.71073	0.71073	0.71073
*a* Å	16.520 (13)	8.226 (1)	8.192 (2)	14.496 (2)	10.038 (1)
*b* Å	6.016 (5)	10.393 (1)	9.112 (2)	9.151 (1)	12.880 (2)
*c*/Å	19.113 (12)	10.544 (3)	12.316 (2)	27.067 (3)	14.011 (2)
*α*/^0^	90	70.54(1)	101.66(1)	90	90
*β*/^0^	113.52 (4)	80.39 (1)	106.90 (1)	105.16 (1)	110.59 (1)
*γ*/^0^	90	84.66 (1)	94.78 (1)	90	90
*V* Å^−3^	1742.0 (2)	838.4 (1)	851.5 (2)	3465.6 (7)	1695.7 (3)
*Z*	4	2	2	8	4
*D*_c_/g cm^−3^	1.386	1.440	1.418	1.393	1.424
*µ*, mm^−1^	0.325	0.338	0.322	0.327	0.334
2*θ* max [°]	48.00	54.38	54.23	54.60	54.38
Limiting indices	−18 ≤ *h* ≤ 18	−10 ≤ *h* ≤ 10	−10 ≤ *h* ≤ 10	−18 ≤ *h* ≤ 18	−12 ≤ *h* ≤ 10
	−6 ≤ *k* ≤ 6	−12 ≤ *k* ≤ 13	−11 ≤ *k* ≤ 11	−9 ≤ *k* ≤ 11	−16 ≤ *k* ≤ 16
	−21 ≤ *l* ≤ 21	−13 ≤ *l* ≤ 13	−15 ≤ *l* ≤ 15	−34 ≤ *l* ≤ 34	−18 ≤ *l* ≤ 18
*F* (000)	760	380	380	1520	760
Total reflections	11 631	15 602	16 636	33 053	45 346
Unique reflections	2724	3709	3743	3888	3763
Reflection at *I* > 2*σ* (*I*)	1704	3538	2691	3227	2788
No. of parameters	226	222	244	218	231
*R*_1_, *I* > 2*σ* (*I*)	0.0595	0.0334	0.0458	0.0363	0.0410
*wR*_2_ *I* > 2*σ* (*I*)	0.1474	0.0859	0.1115	0.0883	0.1175
GooF on F^2^	1.004	1.016	1.016	1.030	1.046
CCDC no.	2292145	2292147	2292146	2292148	2292144

a Due to the disorder of some atoms, the *M*_r_ value is different for Forms II, IV and V.

**Table 2:** Characteristic hydrogen-bond distances (Å) and angles (°) of Forms I–V^a^

Co-crystals	N–H⋯O			N–H⋯N			N–H⋯S			C–H⋯O			C–H⋯N			C–H⋯S		
Form I	1.64	2.650 (5)	175				2.37	3.373 (5)	176	2.57	3.318 (5)	126	2.50	3.207 (6)	122			
	2.08	3.051 (5)	161							2.34	3.365 (6)	157						
Form II	1.87	2.840 (1)	161	1.69	2.687 (1)	170				2.42	3.077 (2)	118						
	1.95	2.934 (1)	165															
Form III	1.93	2.924 (3)	166	1.72	2.731 (4)	176	2.46	3.419 (3)	158	2.30	3.086 (4)	128						
Form IV	1.74	2.715 (2)	162	1.81	2.796 (4)	166	2.49	3.464 (3)	162	2.44	3.287 (5)	134	2.49	3.114 (2)	115	2.73	3.796 (2)	169
	1.97	2.938 (2)	160							2.33	3.358 (2)	158				2.74	3.764 (2)	157
	2.03	2.965 (2)	153							2.41	3.191 (2)	128						
Form V	2.08	2.938 (3)	141	1.79	2.780 (3)	165				2.38	3.352 (3)	149						
	1.93	2.864 (3)	153															

a For each form, the three columns represent the distances of H⋯A and D⋯A and angle ∠D–H⋯A.

#### Powder X-ray diffraction (PXRD) studies

PXRD measurements were carried out utilizing a Bruker D8 Advance X-ray diffractometer equipped with Cu-Kα radiation (*λ* = 1.5418 Å). The applied voltage and current were set at 40 kV and 40 mA, respectively. Samples were subjected to reflection-mode measurements within the 2*θ* range of 5°–40°. The scan speed employed was 15° min^−1^, with a step size of 0.02° and a step time of 0.1 s.

#### Variable-temperature powder X-ray diffraction (VTPXRD) technique

Using the same PXRD equipment, variable-temperature PXRD investigations were conducted. The standard stage was replaced with an Anton Paar TTK450 high-temperature sensitive stage. The ground samples were scanned within an angular span of 2*θ* = 5°–40°. The scanning rate employed was 0.8°, while the temperature was progressively increased from ambient to just prior to the melting temperature, with 5° intervals. To verify the homogeneity and phase alterations, the obtained pattern was compared with the simulated pattern of the single crystal.

#### Differential scanning calorimetry (DSC)

Thermal analyses were done using the PerkinElmer DSC 8000 instrument. The calibration of the instrument was accomplished using an indium sample, with a sealed empty pan as the reference. In each case, a 2 mg sample was placed in a stainless-steel pan and heated within an N_2_ atmosphere at a scanning rate of 5°/min.

#### Solid-state UV-vis absorption spectroscopy

Solid-state UV-vis spectra were recorded on a Shimadzu UV-visible spectrophotometer equipped with a diffuse reflectance attachment. Finely ground powder samples were placed in the sample holder, and spectra were collected in the wavelength range of 200–800 nm at room temperature, using BaSO_4_ as the background reference.

#### Solid-state photoluminescence (PL) spectroscopy

Solid-state photoluminescence (PL) measurements were carried out on a HORIBA spectrofluorometer (Fluorolog-QM), with a xenon lamp as the excitation source. The emission spectra of the powdered samples were recorded at room temperature under the desired excitation wavelength.

### DFT calculations

#### HOMO–LUMO molecular orbital calculations

An ORCA 4.2.1 quantum chemistry package at the density functional theory (DFT) level employing the B3LYP functional with the def2-TZVP basis set was used to compute the HOMO–LUMO energy gap.^49,50^ The calculations were carried out using the experimental crystal geometries obtained from single-crystal X-ray diffraction at room temperature, without further geometry optimization.

#### Lattice energy calculations

The Biovia Material Studio 2020 (version 20.1.0.5)^51^ was utilized to calculate the lattice energy for all the polymorphs. The lattice energy for each polymorph was determined using eqn (1).^52^

1 *E*_latt_ = *E*_bulk_/*Z* − *E*(*A*) − *E*(*B*),

where *E*_bulk_ is the total energy of the optimized crystal structure, and *E*_*A*/*B*_ is the energy of an isolated molecular co-former extracted from the corresponding crystal structure. The calculations were performed using the Dmol3 module with the GGA-PW91 functional.^53,54^

#### Hirshfeld surface analysis

Hirshfeld surface calculation was performed using Crystal Explorer 21.5 software.^55^ All Hirshfeld surfaces were generated using standard high-resolution parameters and plotted as a function of the (*d*_i_, *d*_e_) pair.

## Results and discussion

Mining the Cambridge Structural Database (CSD) revealed that PA, despite being a popular drug molecule, only had twelve co-crystals, as reported in the literature.^54,56^ Thus, to further investigate its propensity to form co-crystals with a variety of complementary molecules, for example, GRAS compounds, the co-crystallization of PA with SA, an artificial sweetener, was carried out, considering the ability of SA to form co-crystals with various drug targets.^57–59^

**Chart 1:**
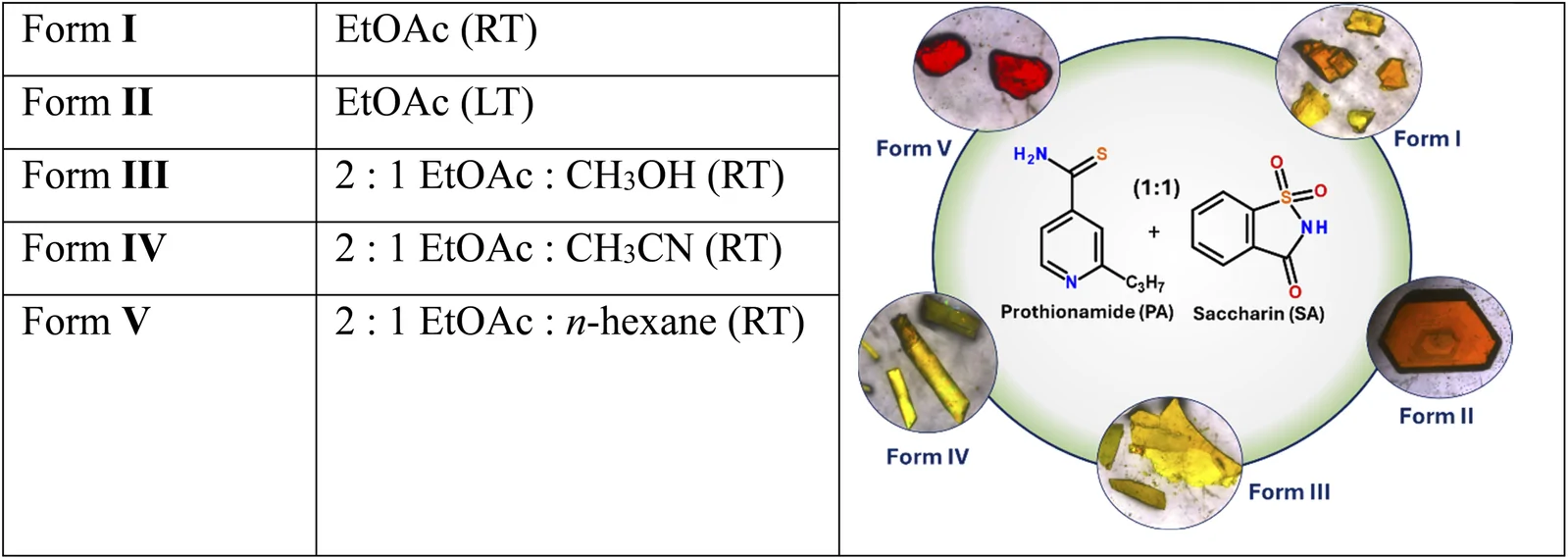
Crystallization details and morphology of the Forms I–V.

Upon the co-crystallization of PA and SA in a 1 : 1 ratio, five different crystalline forms were obtained, depending on the crystallization solvent and preparation conditions (temperature), as illustrated in Chart 1, while fully conserving the ratio of the co-formers. The five forms are labelled I–V to further discuss the structural and other solid-state features. Forms I and II were obtained from an EtOAc solution by slow evaporation at ambient and low temperatures (5–10 °C), respectively. The impact of solvent polarity on crystallization, which is often modulated by solvent mixtures, was considered. Likewise, the crystallization of PA and SA was carried out using a polar and non-polar solvent mixture consisting of CH_3_OH/CH_3_CN/*n*-hexane added at 20% (v/v) to EtOAc, affording Forms III–V. Forms I–V were distinguishable by different colours and distinct morphology (see Chart 1). Thus, while Forms I and III appeared as orange blocks and yellow plates, Forms II, IV and V grew as red hexagonal, yellow rod and red rectangular crystals, respectively. All crystal forms were characterized and analysed through a range of analytical techniques, including single-crystal X-ray diffraction (SCXRD), variable-temperature powder X-ray diffraction (VTPXRD), and differential scanning calorimetry (DSC). Further, the structural characterization of the novel polymorphs was investigated through conformational analysis and lattice energy calculation to determine their molecular orientations and thermodynamic stability. Additionally, Hirshfeld surface analysis was utilized to quantify the strength and nature of the intermolecular interactions governing the crystal packing. The complete details of these features are discussed in the following sections, beginning with the packing of molecules in the crystal lattices of each form, providing the relevant crystal data and characteristics of intermolecular interactions in Tables 1 and 2, respectively.

### Crystal-structure analysis of polymorphs I–V

**Fig. 1:**
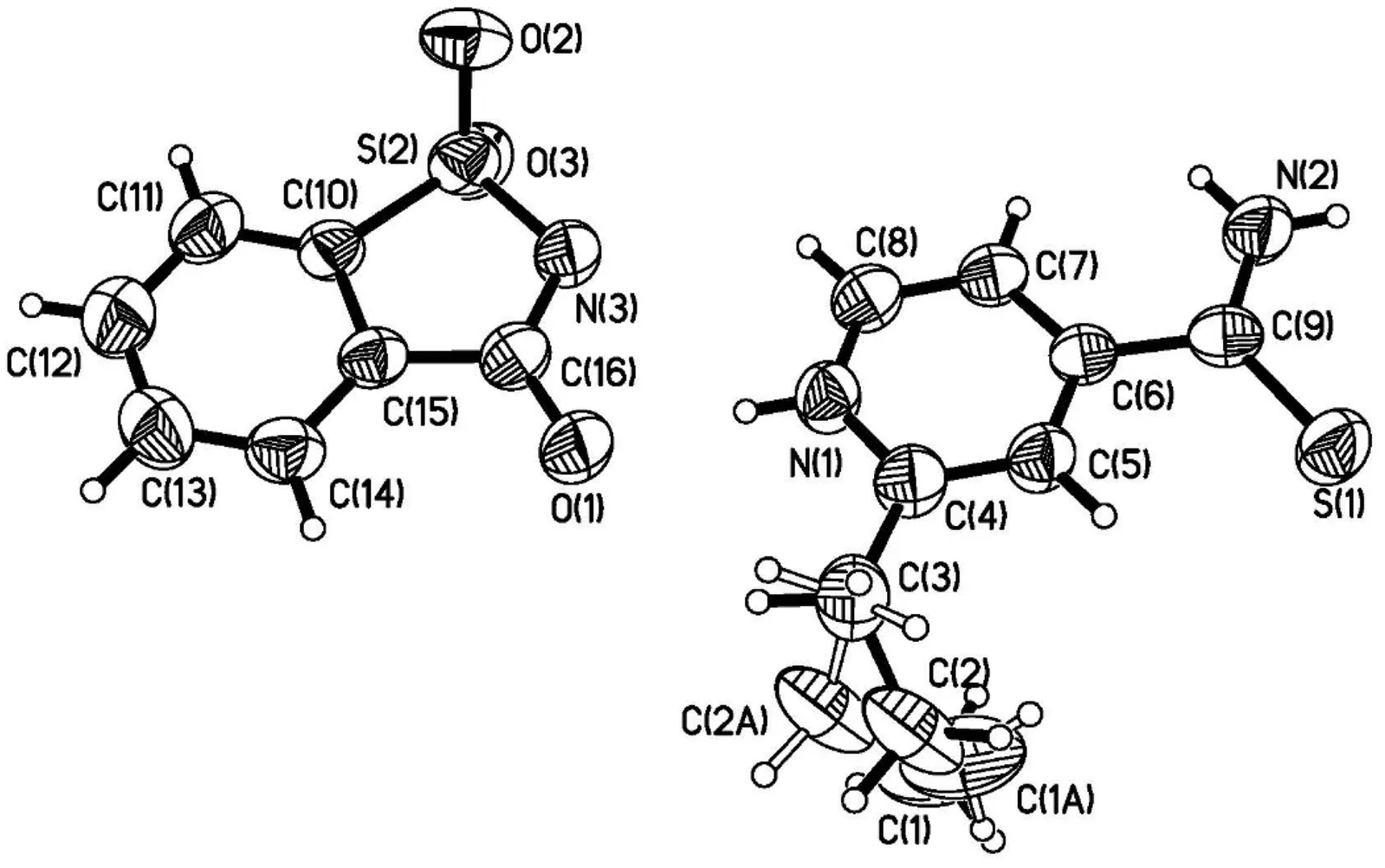
ORTEP diagram of the asymmetric unit in the crystal structure of Form I.

Structural determination by single-crystal X-ray diffraction studies confirmed a consistent 1 : 1 asymmetric unit in all polymorphs, with distinct cell parameters, space groups and hydrogen-bonded motifs that define the polymorphic diversity among the forms. A typical asymmetric unit is shown in Fig. 1 for Form I, as an ORTEP representation, while all other forms are shown in the SI (see Fig. S1–S4). All the relevant crystallographic information is given in Table 1.

#### Form I

**Fig. 2:**
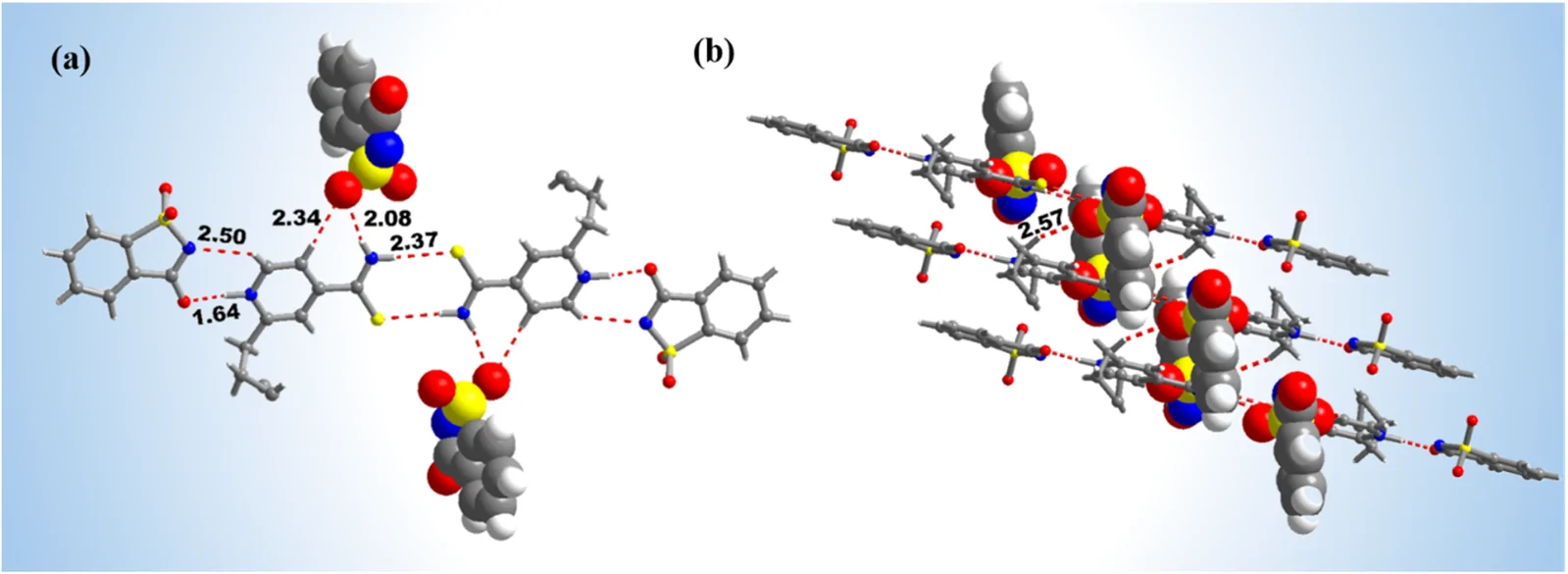
(a) Recognition pattern within a chain arrangement. (b) Propagation of chains along *c*-axis.

**Fig. 3:**
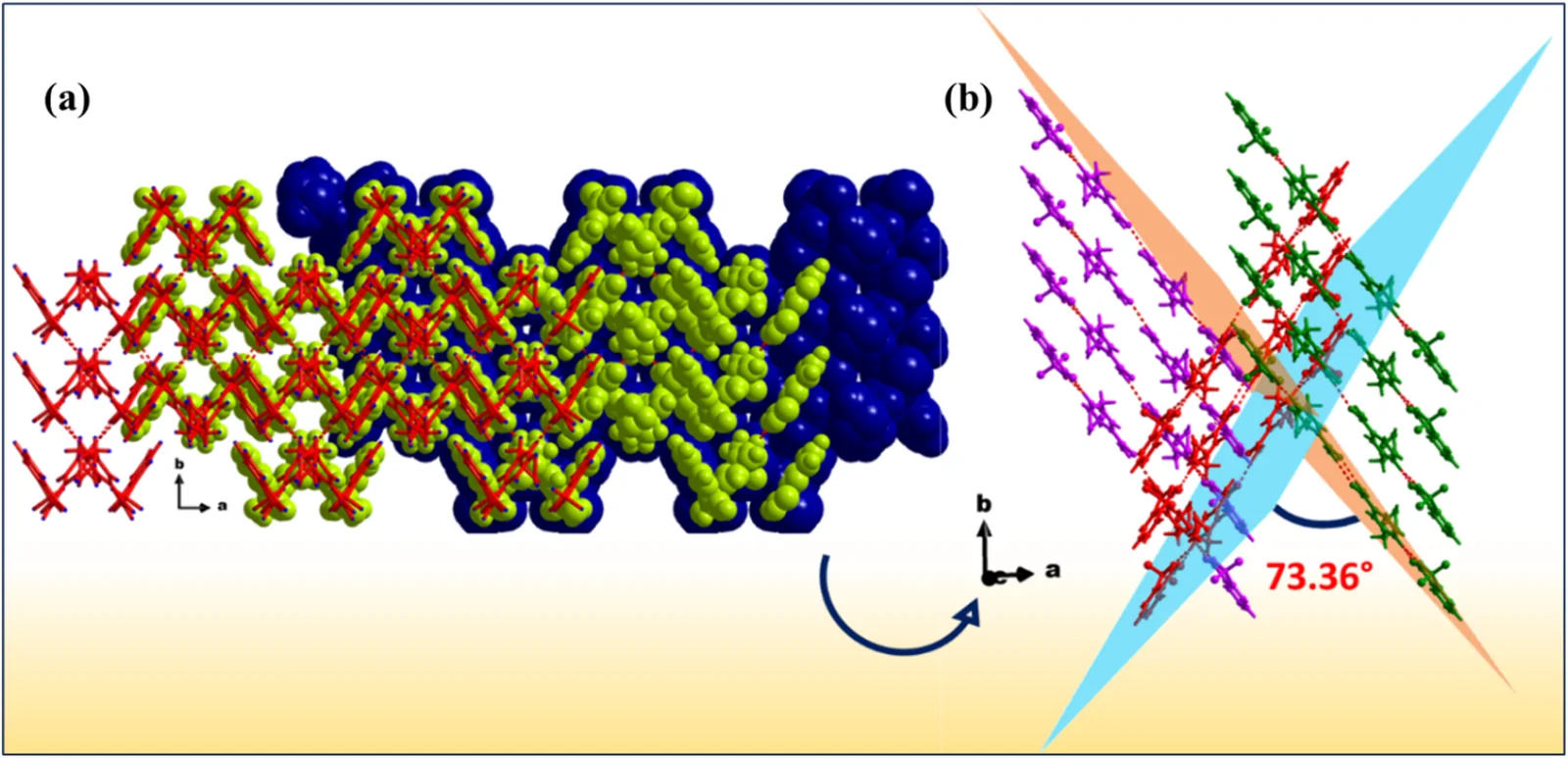
(a) Zigzag-layered arrangement in Form I. (b) Typical two-dimensional tape arrangement.

The molecular recognition analysis of the crystal lattice of Form I, which crystallized in a monoclinic *P*2/*n* space group, revealed that the PA molecules assembled into centrosymmetric *R*_2_^2^ (8) homomeric dimers *via* N–H⋯S hydrogen bonds (H⋯S, 2.37 Å; N⋯S, 3.37 Å). These dimers were subsequently glued to four surrounding SA molecules through two types of cyclic bonds (Fig. 2a). One of the cyclic pattern comprised pair-wise N^+^–H⋯O (H⋯O, 1.64 Å; N^+^⋯O, 2.65 Å) and C–H⋯N^−^ (H⋯N^−^, 2.50 Å; C⋯N^−^, 3.20 Å) hydrogen bonds, while the other one was due to N–H⋯O and C–H⋯O (H⋯O, 2.08 Å; N⋯O, 3.05 Å and H⋯O, 2.34 Å; C⋯O, 3.36 Å) hydrogen bonds. Thus, the SA molecules interacted with the PA molecules, both in parallel and perpendicular directions to the plane of the PA dimers, as depicted in Fig. 2a, due to the angular orientation of an –SO_2_ group. Intriguingly, the perpendicularly oriented SA chains, interlinked by a C–H⋯O (H⋯O, 2.57 Å; C⋯O, 3.32 Å) hydrogen bond (as shown in Fig. 2b), ultimately yielded a 3D zigzag layered structure of 73.36° entangled corrugated tapes (Fig. 3).

#### Form II

Form II crystallized in a triclinic *P*1̄ space group, with a 1 : 1 molecular composition in the asymmetric unit, thereby establishing a polymorphic relation with Form I. Interestingly, in both Forms (I and II), the propyl chain showed high thermal displacement parameters, but it was well resolved into an acceptable range in Form II upon carrying out the structure determination using the X-ray intensity data collected at low temperature (100 K), as shown in Fig. S1.

**Fig. 4:**
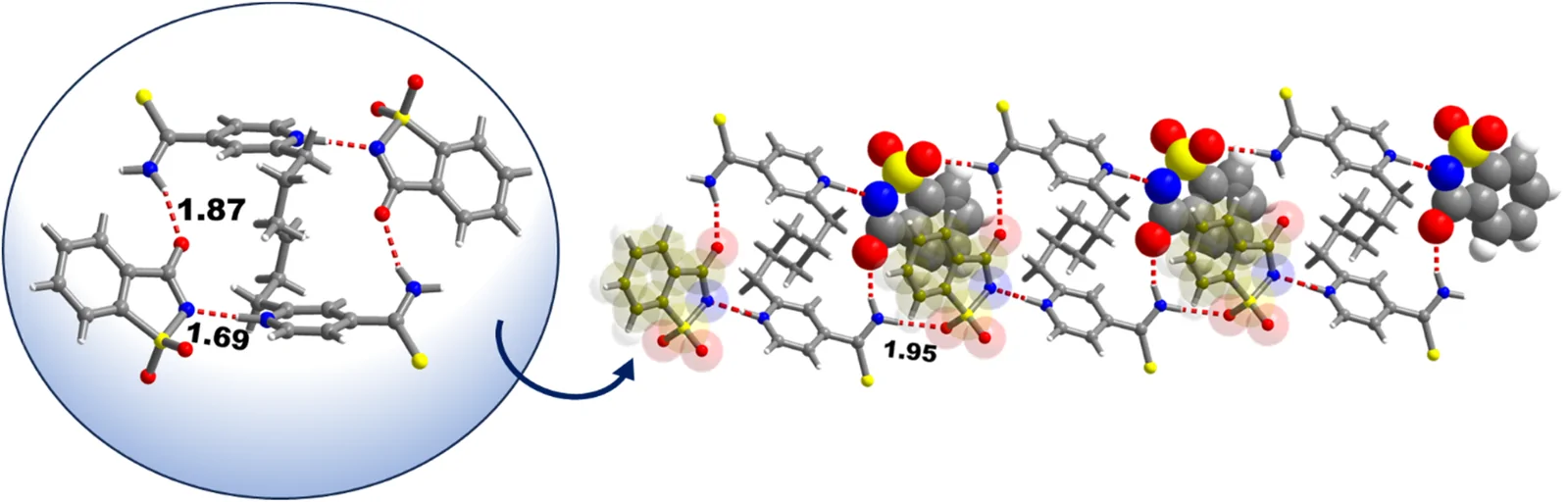
Four-membered cyclic rings formed between PA and SA in Form II.

Structural analysis showed that four-membered cyclic rings were realized in Form II by involving two molecules each of PA and SA in an alternating way through the formation of N–H⋯O (H⋯O, 1.87 Å; N⋯O, 2.84 Å) and N^+^–H⋯N^−^ (H⋯N^−^, 1.69 Å; N^+^⋯N^−^, 2.69 Å) hydrogen bonds. While the former ones were due to the interaction between –NH_2_ of PA and the carbonyl moiety of SA, the latter was formed between pyridyl-*N* of PA and ring-*N* of SA. Furthermore, the cyclic four-membered rings were held together and propagated to form an infinite double-chain structure through the formation of N–H⋯O (H⋯O, 1.95 Å; N⋯O, 2.93 Å) hydrogen bonds. The pictorial representation of the molecular recognition and formation of chains in Form II is shown in Fig. 4.

**Fig. 5:**
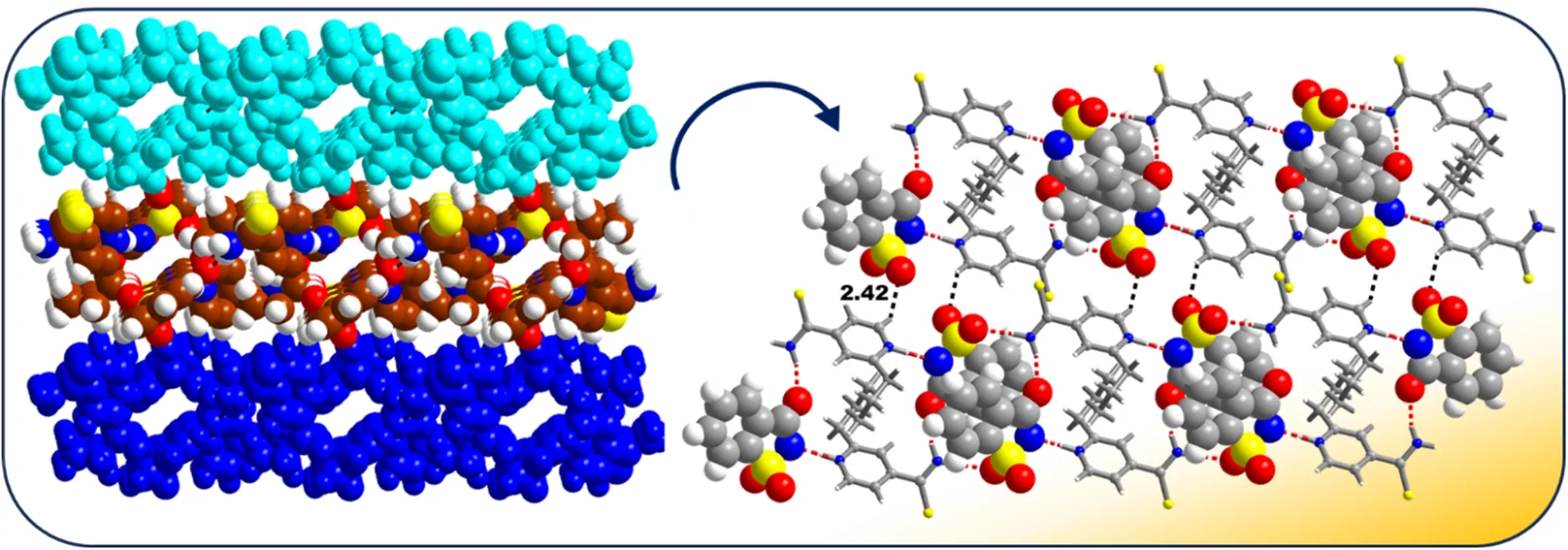
Three-dimensional arrangement of constituent molecules (co-formers) in Form II.

Such molecular chains were further packed in a three-dimensional arrangement, as presented in Fig. 5, culminating in the form of stacked layers by holding the adjacent layers through C–H⋯O (H⋯O, 2.42 Å; C⋯O, 3.08 Å) hydrogen bonds.

#### Form III

Form III, obtained upon dissolving the co-formers in a solvent mixture (2 : 1 EtOAc and MeOH), crystallized as a polymorph of Forms I and II with the same asymmetric unit (an equal molar ratio of the co-formers).

**Fig. 6:**
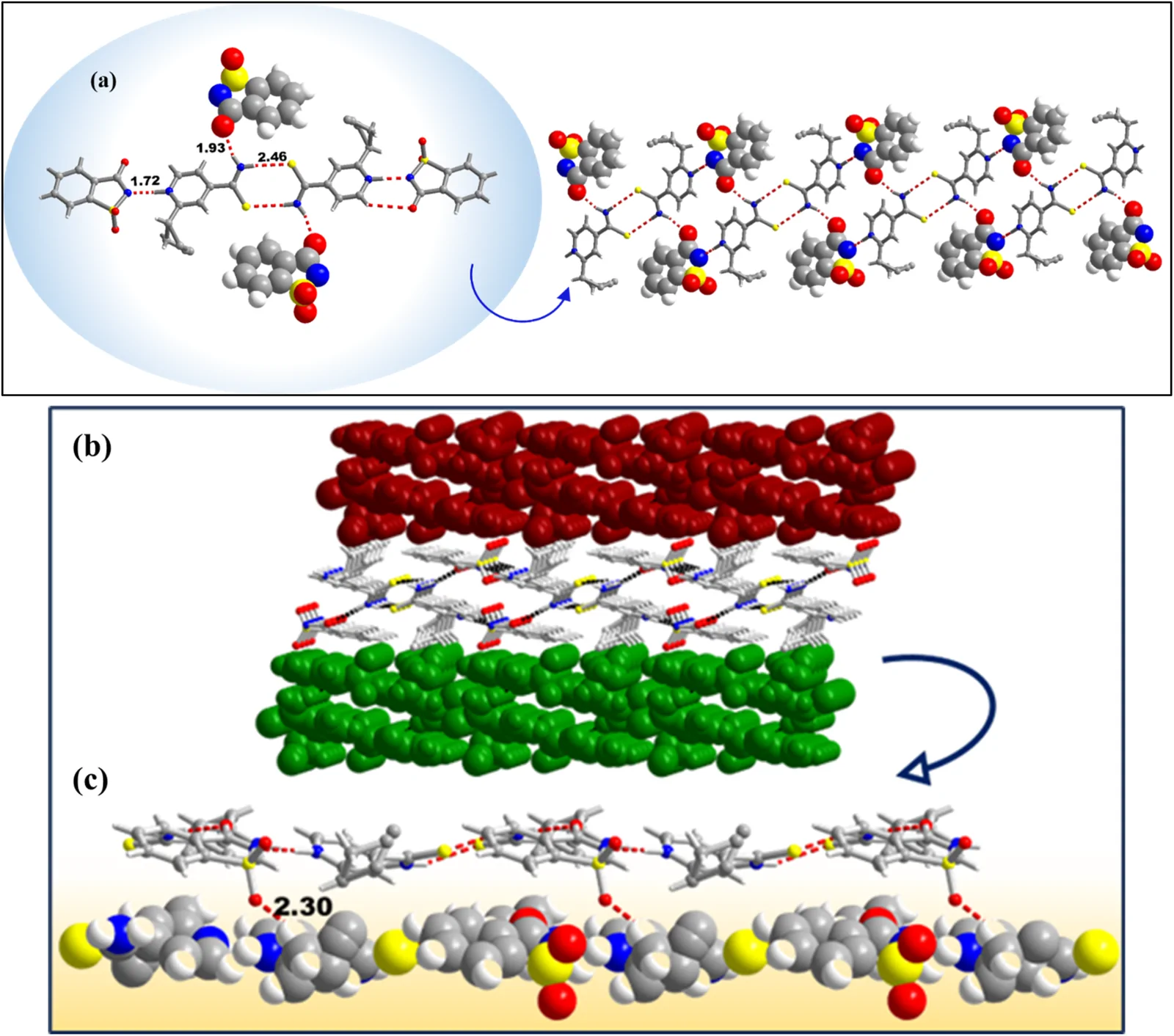
(a) Basic recognition pattern between the co-formers in the crystal structure of Form III. (b) Three-dimensional arrangement of constituents in Form III. (c) Two adjacent tapes interact through the C–H⋯O hydrogen bonds.

The analysis of the recognition and packing features revealed that the PA molecules formed homomeric dimers through the *R*_2_^2^ (8) pattern, formed by the N–H⋯S hydrogen bonds (H⋯S, 2.46 Å; N⋯S, 3.42 Å). In turn, these dimers were held together by SA molecules through acyclic N^+^–H⋯N^−^ and N–H⋯O hydrogen bonds (H⋯N^−^, 1.72; N^+^⋯N^−^, 2.73 and H⋯O, 1.93 Å; N⋯O, 2.92 Å), involving both the –NH_2_ and pyridyl-*N* atoms of PA and the hetero N-atom and –C=O group of SA (Fig. 6a). Furthermore, this moiety propagated to constitute linear layers in a two-dimensional arrangement, which were stacked in a three-dimensional arrangement (see Fig. 6b), establishing C–H⋯O hydrogen bonds (H⋯O, 2.30 Å; C⋯O, 3.09 Å) between the adjacent layers, as shown in Fig. 6c.

#### Form IV

Form IV was obtained by changing the solvent mixture to EtOAc/CH_3_CN, replacing the more polar MeOH co-solvent with the less polar acetonitrile, resulting in a monoclinic *C*2/*c* space group with distinctly different unit cell parameters (Table 1), but with the same asymmetric unit content as Forms I–III.

**Fig. 7:**
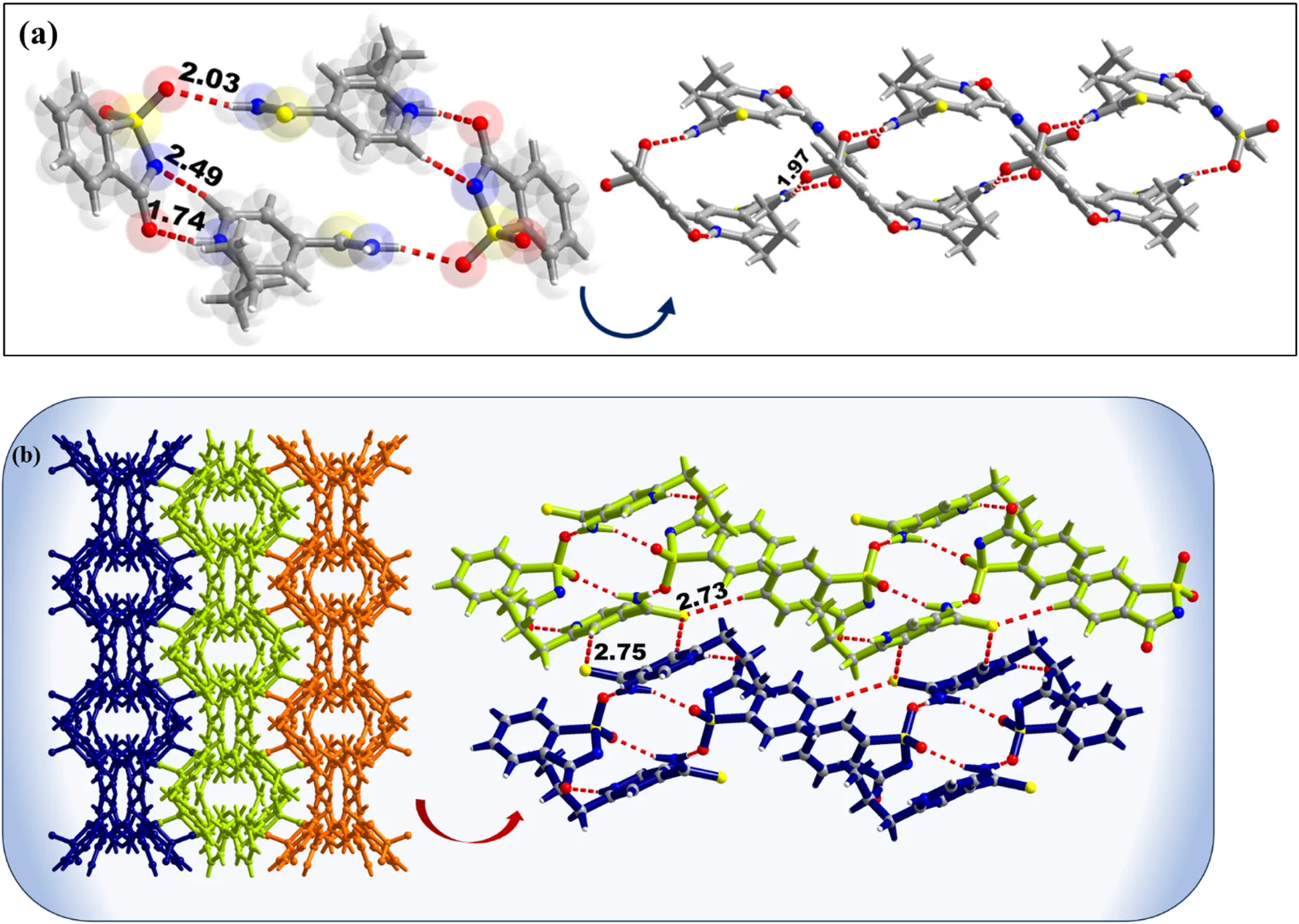
(a) Basic recognition pattern (in the form of rings) and propagation of the rings in the crystal structure of Form IV. (b) Arrangement of the molecules in Form IV in three and two dimensions.

In Form IV, the PA and SA molecules formed heteromeric hydrogen bonding in the form of *R*_2_^2^ (8) and an acyclic *C*_1_^1^ (2). The cyclic pattern involved N^+^–H⋯O (H⋯O, 1.74 Å; N^+^⋯O, 2.72 Å) and C–H⋯N^−^ (H⋯N^−^, 2.49 Å; C⋯N^−^, 3.12 Å) hydrogen bonds, whereas the acyclic motif was established by N–H⋯O (H⋯O, 2.03 Å; N⋯O, 2.97 Å) hydrogen bonds. Such adjacent rings were held together by N–H⋯O (H⋯O, 1.97 Å; N⋯O, 2.94 Å) hydrogen bonds. The association of molecules in this arrangement is highlighted in Fig. 7a. Further, in a three-dimensional arrangement, the structure was stabilized by a stacked layered arrangement through C–H⋯S (H⋯S, 2.75 Å; C⋯O, 3.77 Å) hydrogen bonds (see Fig. 7b). In fact, this represents the first instance where the involvement of S atoms in the formation of interactions is observed.

#### Form V

The red crystals of polymorph V were obtained from a solvent mixture of EtOAc/*n*-hexane by replacing the polar co-solvents, MeOH (Form II) and CH_3_CN (Form IV), with non-polar *n*-hexane. Form V was crystallized with the same asymmetric unit contents as observed in Forms I–IV, but with distinctly different unit cell dimensions (Table 1).

**Fig. 8:**
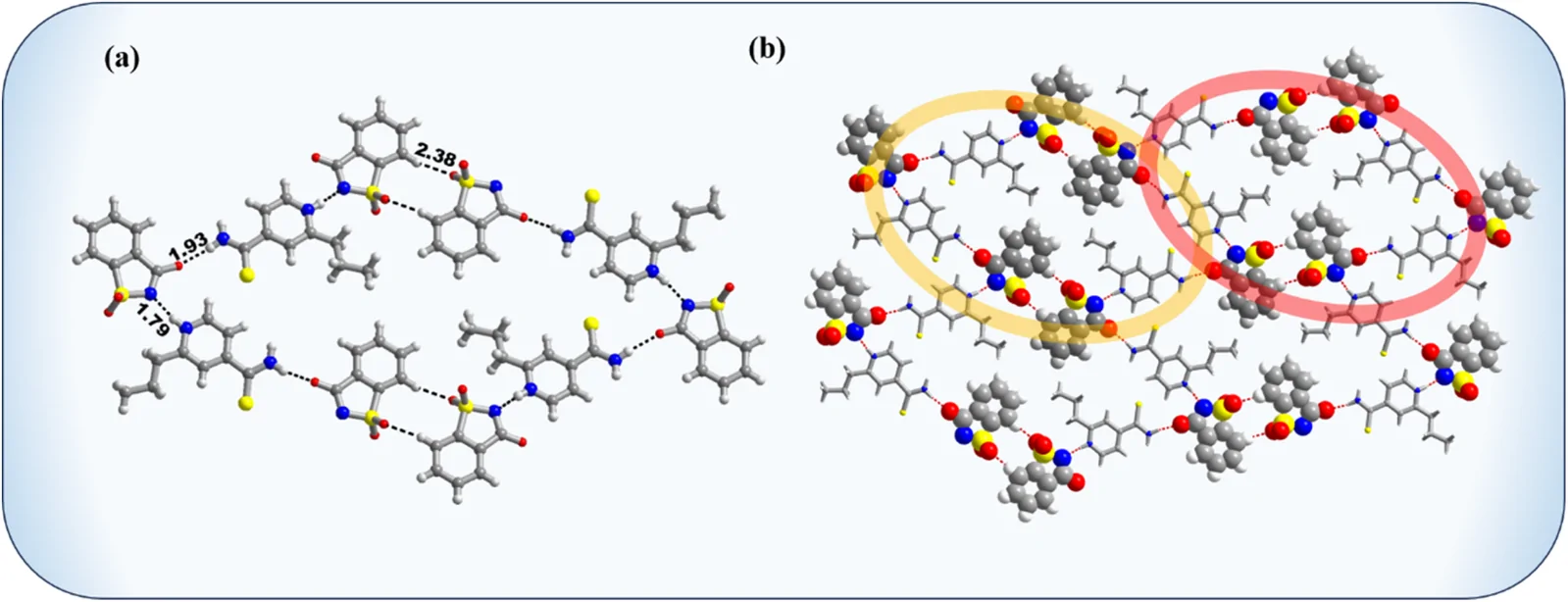
(a) Ten-membered cyclic ensembles formed through the primary interactions between PA and SA. (b) 2D sheet arrangement of Form V.

Crystal structure analysis revealed that adjacent SA molecules were held together as dimers by centrosymmetric cyclic C–H⋯O (H⋯O, 2.38 Å; C⋯O, 3.35 Å) hydrogen bonds. Such dimers were further connected through a PA molecule with the aid of two different types of hydrogen bonds, N–H⋯O (H⋯O, 1.93 Å; N⋯O, 2.86 Å) and N^+^–H⋯N^−^ (H⋯N^−^, 1.79 Å; N^+^⋯N^−^, 2.78 Å). Such an arrangement, thus, constituted a ten-membered cyclic ensemble of the PA and SA molecules in a 4 : 6 ratio, respectively, as shown in Fig. 8a. These ensembles further interacted with each other in the form of a typical sheet-like structural arrangement (see Fig. 8b).

**Fig. 9:**
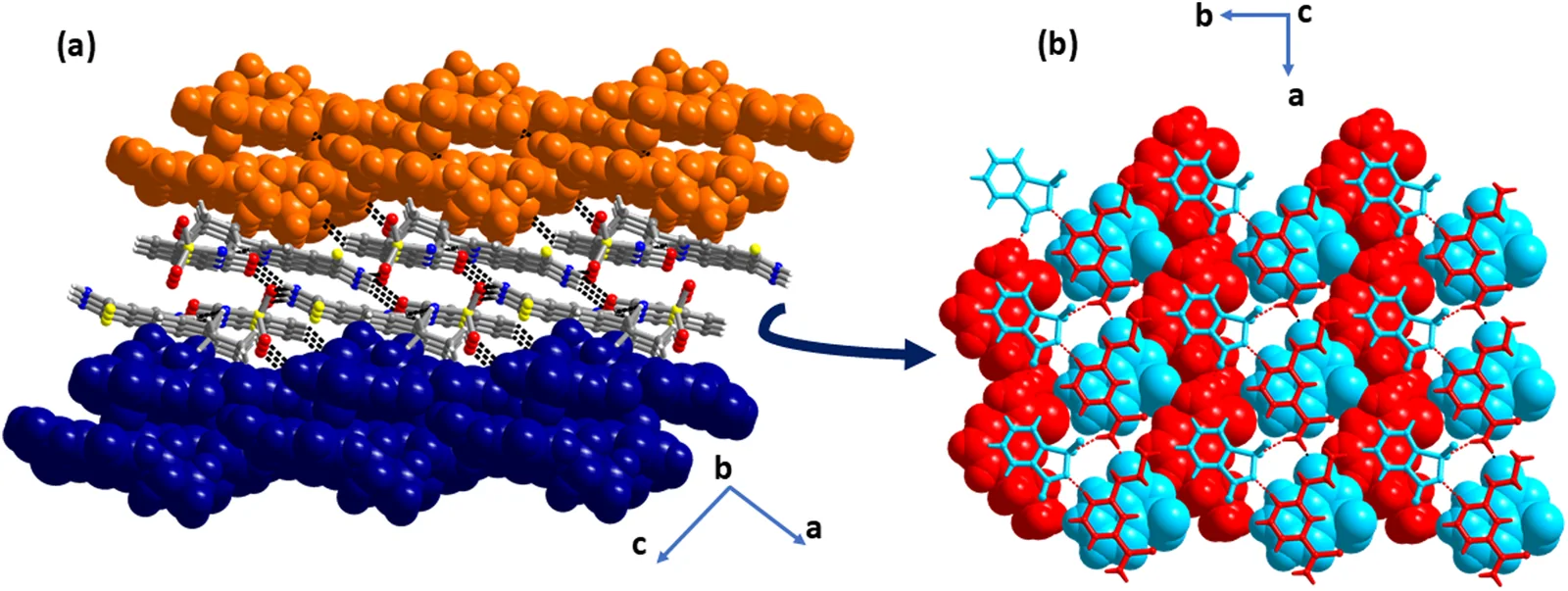
(a) Three-dimensional layer arrangement and (b) representation of two layers along the *c*-axis.

In the three-dimensional arrangement, the sheets combined to form a stacked layered arrangement, and the adjacent layers were further interconnected through C–H⋯O hydrogen bonds. A pictorial representation of the stacking is illustrated in Fig. 9.

### Structural correlation of forms I–V

Co-crystals are the result of molecular recognition between the functional groups of opposite electronic features present on the co-formers. Though the groups show conformational flexibility to form different types of patterns of interactions classified and coded, one of the stable patterns often directed the formation of the co-crystals. For instance, though –COOH is able to form dimeric and catemeric patterns, the former is prevalent in many of the assemblies, perhaps due to the formation of strong O–H⋯O hydrogen bonds. However, for a similar functional moiety like –CSNH_2_, the involvement of weak N–H⋯S hydrogen bonds stabilizes both centrosymmetric and acentric arrays. This strong affinity was apparently responsible for the observed different polymorphs between PA and SA.

**Scheme 1:**
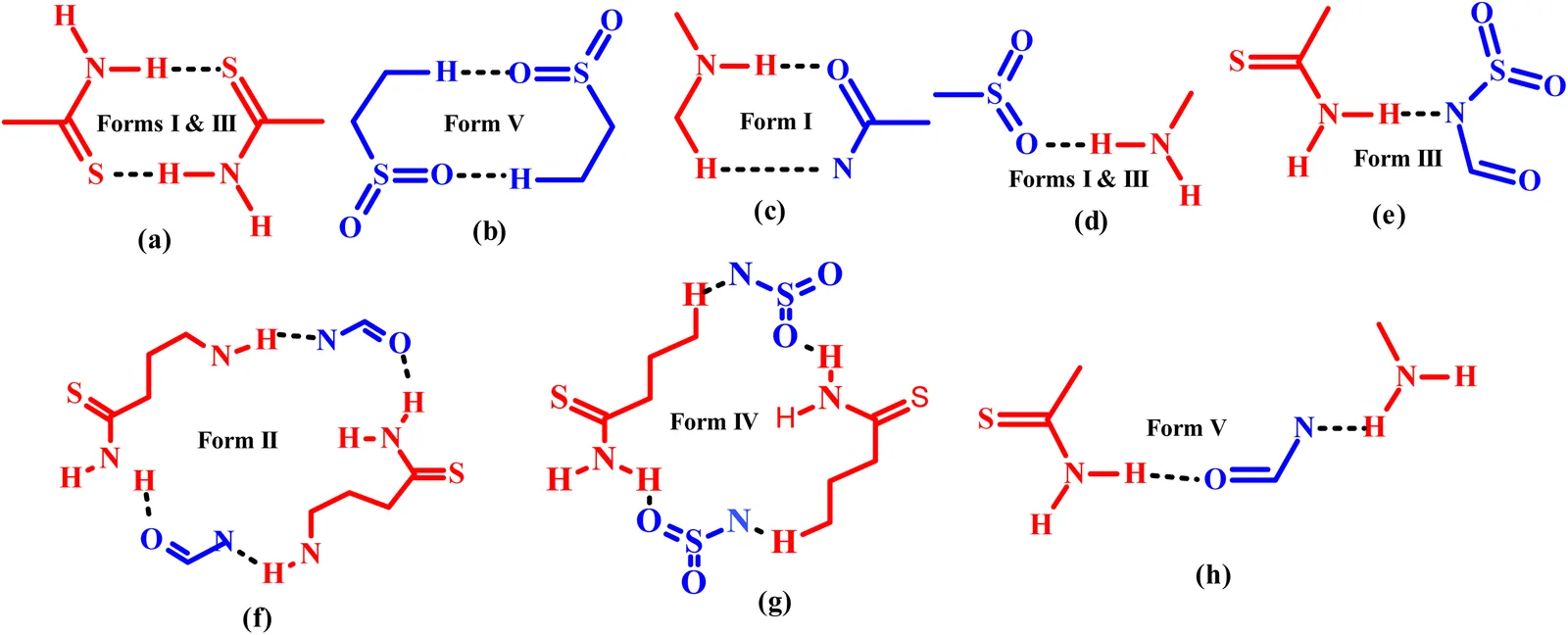
Patterns of the intermolecular interactions in Forms I–V.

At a molecular level, in Forms I and III, a disordered propyl chain was observed; such a chain showed a high degree of thermal vibration in Form II; however, in Forms IV and V, the molecules were fully ordered. However, with reference to the molecular packing in the crystal lattices, a collective structural analysis revealed that the five polymorphs of the co-crystals of PA and SA (Forms I–V) showed heteromeric hydrogen bonding between the co-formers through a cyclic/acyclic pattern, as shown in Scheme 1(c–h). The moieties corresponding to each co-former in the skeletal presentation are represented in different colours – PA (red) and SA (blue). Besides, interestingly, one of the co-formers in Forms I, III and V existed (see Scheme 1(a and b)) as homomeric pairs (dimers) through the formation of centrosymmetric cyclic hydrogen-bonding patterns (Scheme 1a and 1b). In Forms I and III, such a pattern evolved due to the PA molecules, while the SA molecules showed such an association in Form V. Among all, Forms II and IV formed exclusively cyclic heteromeric moieties (Scheme 1(f and g)), comprising four molecular units (two from each PA and SA), with the former exhibiting different types of hydrogen bonds. Among the acyclic patterns, while Forms I–III formed binary units (Scheme 1(d and e)), Form V formed a trimeric unit (Scheme 1h).

The fundamental structural divergence among Forms I–III lay in the pattern of interaction between the SA and PA molecules; such interaction in Form III appeared to be a hybrid of the interactions and topologies observed in Forms I and II. In Form III, the PA molecules formed a centrosymmetric homomeric topology promoted by the N–H⋯S hydrogen bonds between the adjacent molecules and glued to four SA molecules. However, it mimicked the topology observed in Form II, with the establishment of a cyclic topology comprising four molecules (two each of PA and SA), in which SA interacts through the carbonyl oxygen (–C=O) and the ring nitrogen atom with the –NH_2_ and pyridyl-*N* of PA.

Considering the varied structural features deduced using X-ray intensity data and the disclosure of similarities and differences among the forms, further qualitative and quantitative investigations were carried out to assess the stability of the complexes under ambient and non-ambient conditions, as well as the efficacy of intermolecular interactions (primarily hydrogen bonds).

### Differential scanning calorimetry (DSC) analysis

**Fig. 10:**
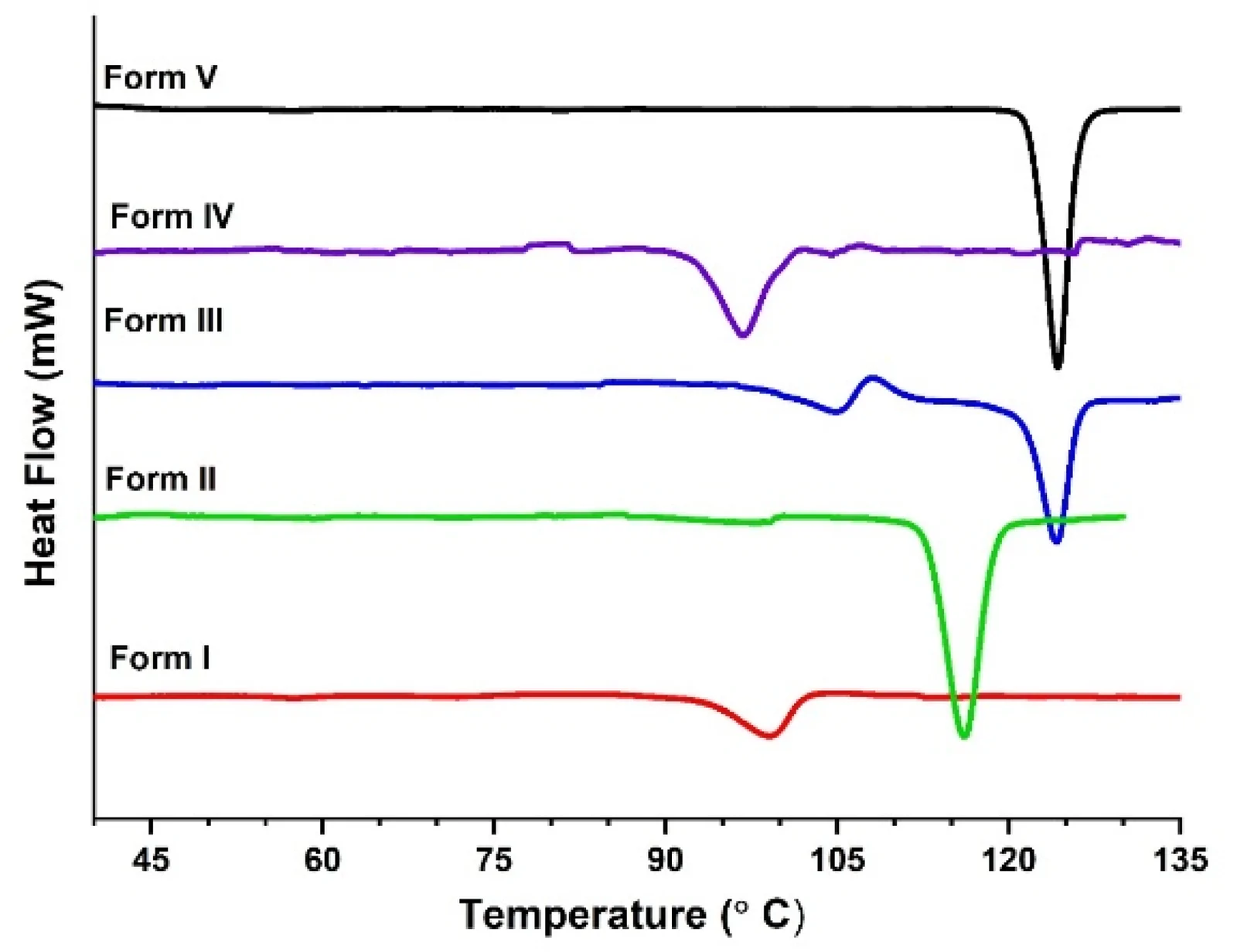
DSC thermogram of Forms I–V.

Thermal analysis studies aimed at evaluating the stability of all the co-crystal polymorphs (I–V) interestingly revealed other silent features: co-crystal melting points (m.p.) lower than the m.p. of either of the co-formers (Table 3) and putative solid-state transformations. Besides, all the forms (I–V) were found to be stable up to the melting point, with only one melting endothermic peak. However, Form III showed two endothermic peaks at 97 and 118 °C; the second endotherm matched the melting endotherm of Form V, suggesting that at 97 °C, Form III transformed into Form V. Thus, Form III may be a meta-stable form under non-ambient conditions, as compared to all the observed forms discussed above. The recorded endotherms are compiled in Fig. 10. This phase transformation was further confirmed by a variable-temperature *in situ* powder X-ray diffraction experiment. The different melting points of all forms are listed in Table 3, further showing the decreasing order: Form V > Form II > Form III > Form I > Form IV.

**Table 3:** Melting point of Forms I–V

Forms	Co-former melting point (°C)		*T*_onset_ (°C)	Δ*H*_m_ (J g^−1^)
	PA	SA		
I	137	228	92	99.09
II	137	228	110	116.13
III	137	228	97, 119	14.41 and 124.20
IV	137	228	90	96.72
V	137	228	119	124.29

### *In situ* variable-temperature powder X-ray diffraction (VTPXRD) analysis

**Fig. 11:**
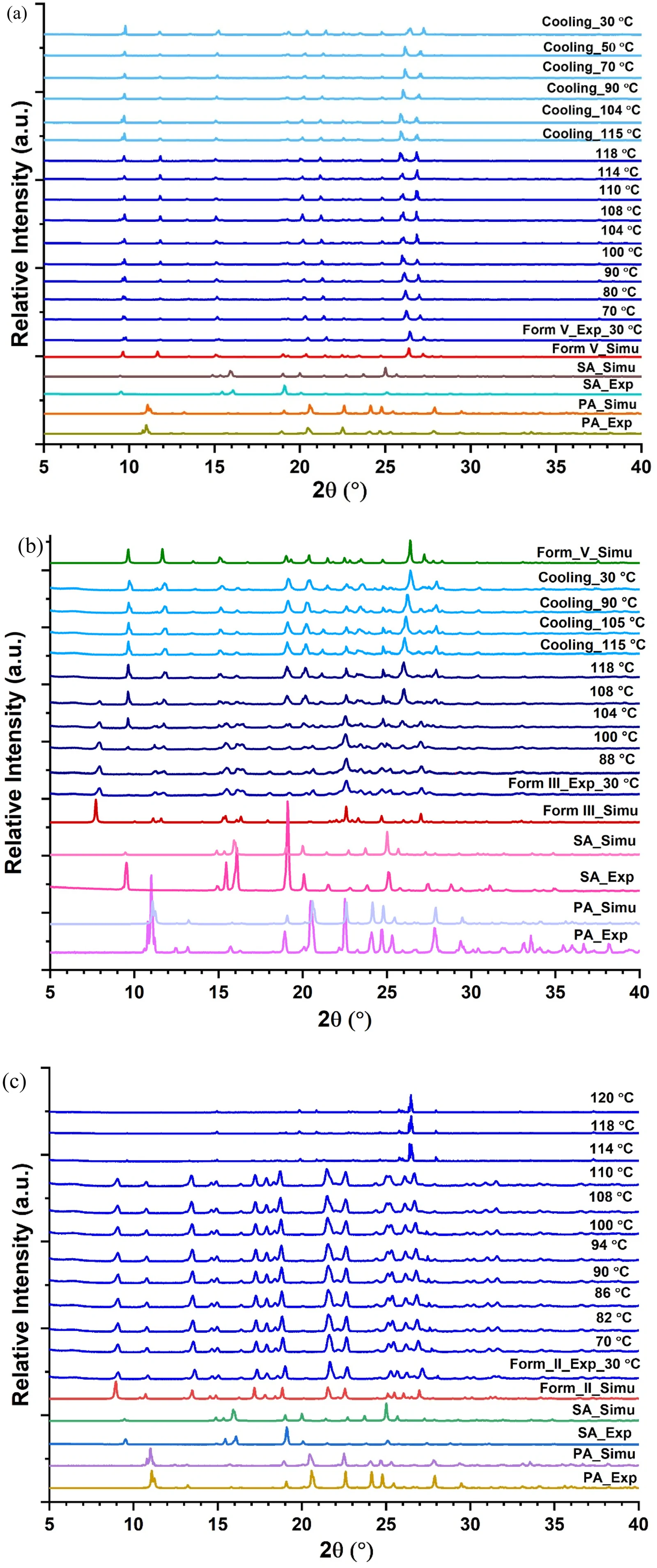
VTPXRD experiment performed on (a) Form V, (b) Form III and (c) Form II.

Considering the transformation of Form III → V qualitatively determined by the DSC method, to evaluate the nature of the transformation and to augment the DSC measurements, *in situ* variable-temperature X-ray diffraction experiments were carried out on ground samples of Forms III and V. The patterns were recorded by heating the samples at a constant temperature gradient, and the observed features are compiled in Fig. 11. The patterns corresponding to Form V (Fig. 11a) remained unchanged throughout the heating range, up to the melting point, confirming the stable features of Form V. However, patterns corresponding to Form III showed new peaks (2*θ*: 9.63°, 11.68°, 26.4°, and 27.24°) in the temperature range of 104–108 °C, which incidentally corresponded to Form V. At the same time, the characteristic peaks of Form III (2*θ*: 7.72°, 11.10°, 11.60°, 22.58°, 24.68°, and 27.02°) disappeared around the same temperature range. The observed changes are shown in Fig. 11b. Thus, the transformation of Form III → V was authenticated unequivocally. However, other forms (I, II, and IV) were noted to be stable during *in situ* heating, as demonstrated by the powder diffraction patterns. A typical pattern for Form II is shown in Fig. 11c. All the characteristic peaks (2*θ*: 8.96°, 13.50°, 17.18°, 18.84°, 21.56°, 22.56° and 26.96°) of Form II were found to remain unchanged throughout the heating process.

### Conformational analysis

**Fig. 12:**
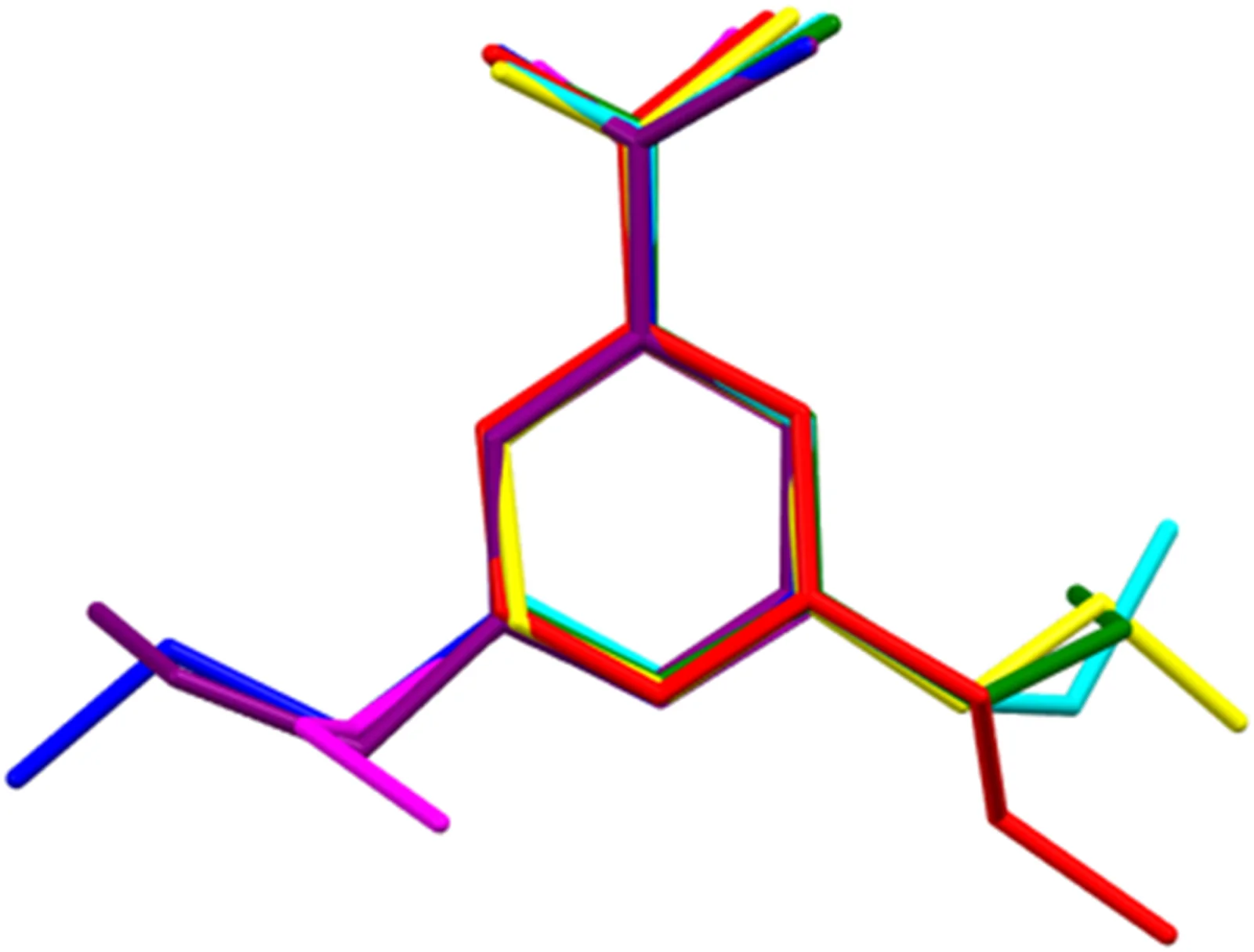
Overlay diagram of the polymorphs of native PA and the co-crystal polymorphs (I–V); PRTCPY20 (red), PRTCPY22 (blue), Form I (green), Form II (purple), Form III (cyan), Form IV (magenta), and Form V (yellow).

Conformational flexibility refers to the ability of organic molecules to adopt different three-dimensional arrangements (conformations) while maintaining the same chemical structure. When co-crystals are formed with co-formers of different functionalities, the potency of conformational variations becomes more significant. Also, the interactions between the co-formers depend on the conformation of molecules and *vice versa*. This interplay between different conformations and intermolecular interactions contributes to the diversity of the co-crystal structures that can be formed. Such an understanding of the factors that account for the variable conformations may facilitate the growth of polymorphs, even in the case of multicomponent systems. Here, in the complex of PA and SA, the existence of flexible functional groups, such as –CSNH_2_, and substituted propyl chains on the molecular structure of PA and the typical interactions with the SA molecule induced numerous conformational arrangements of the PA molecule in those complexes. In fact, native PA existed in two polymorphs clearly distinguished by the different conformations of the propyl group, in *syn* (PRTCPY20) and *anti* (PRTCPY22) positions, with respect to the S atom of the thioamide moiety. This was clearly reflected in the polymorphs (I–V), as represented in the overlay diagram (Fig. 12), along with the native structures of PA; Forms I, III and V were oriented in the *syn* conformation, while Forms II and IV corresponded to the *anti* conformation. The torsional angles around the propyl chain corresponding to the different conformations are listed in Table 4.

**Table 4:** Torsion angles are represented for the native PA molecule, as well as for Forms I–V

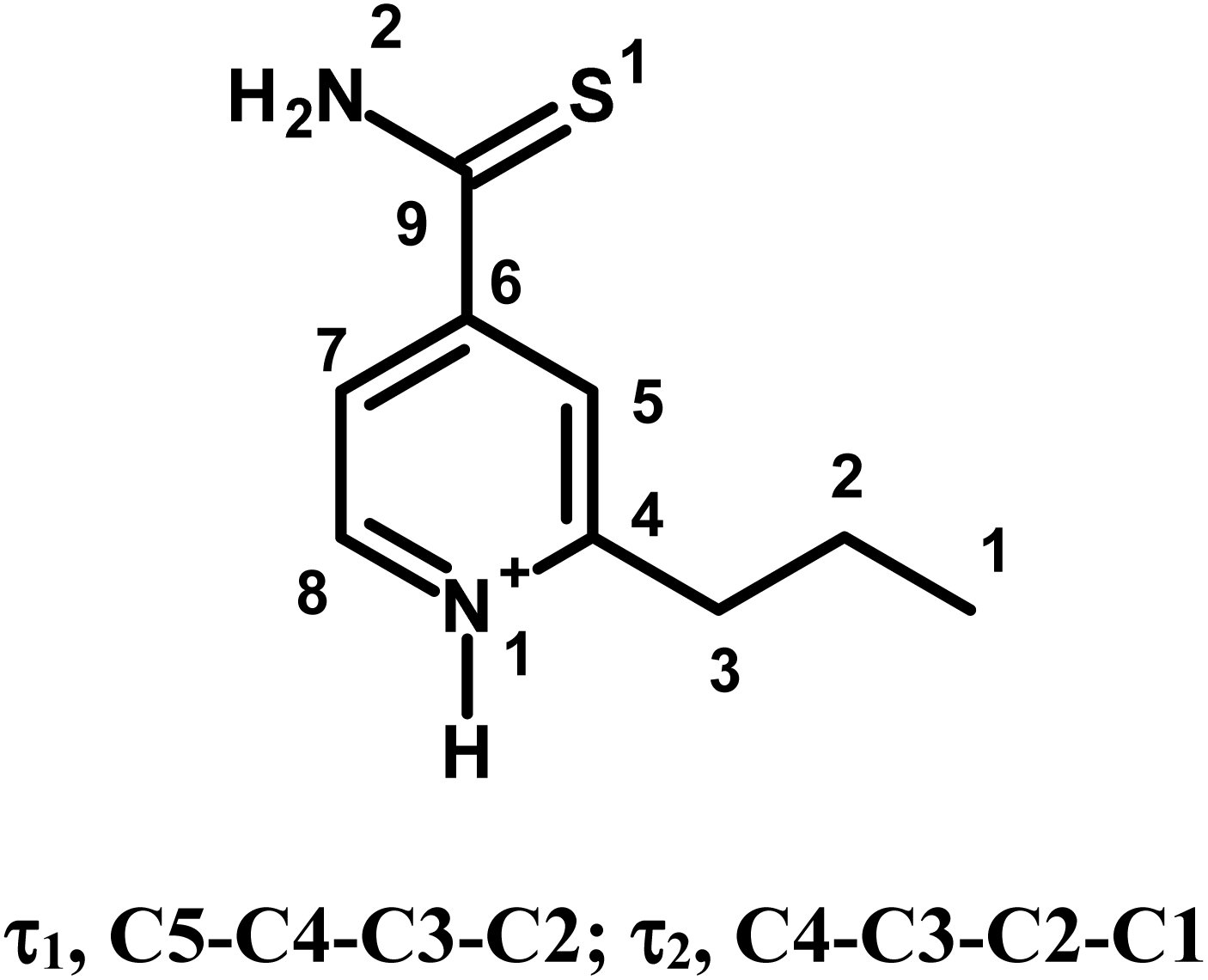			
Complexes	*τ*_1_	*τ*_2_	Orientation
Form I	−46.46	−49.87	*syn*
Form II	21.81	67.01	*anti*
Form III	−92.35	80.82	*syn*
Form IV	−73.84	−70.55	*anti*
Form V	−9.64	−171.11	*syn*
PRTCRY20	−111.98	−173.89	*syn*
PRTCRY22	5.16	177.60	*anti*

A close look at the torsion angles further revealed that between the two polymorphs of native PA, in only PRTCPY20, the propyl chain was out of the molecular plane, with a torsion of 112° (C3–C4). In contrast, in co-crystal polymorph Form V, the propyl chain was within the molecular plane, as observed in PRTCPY22, while in all other forms (I–IV), the chains were out of the molecular plane, with varied torsion angles but a similar magnitude around both C3–C4 and C2–C3 (Form I, 46° and 50°; Form III, 92° and 81°; Form IV, 74° and 71°). However, in Form II, the two torsional angles were different (C3–C4, 22°; C2–C3, 67°).

**Scheme 2:**
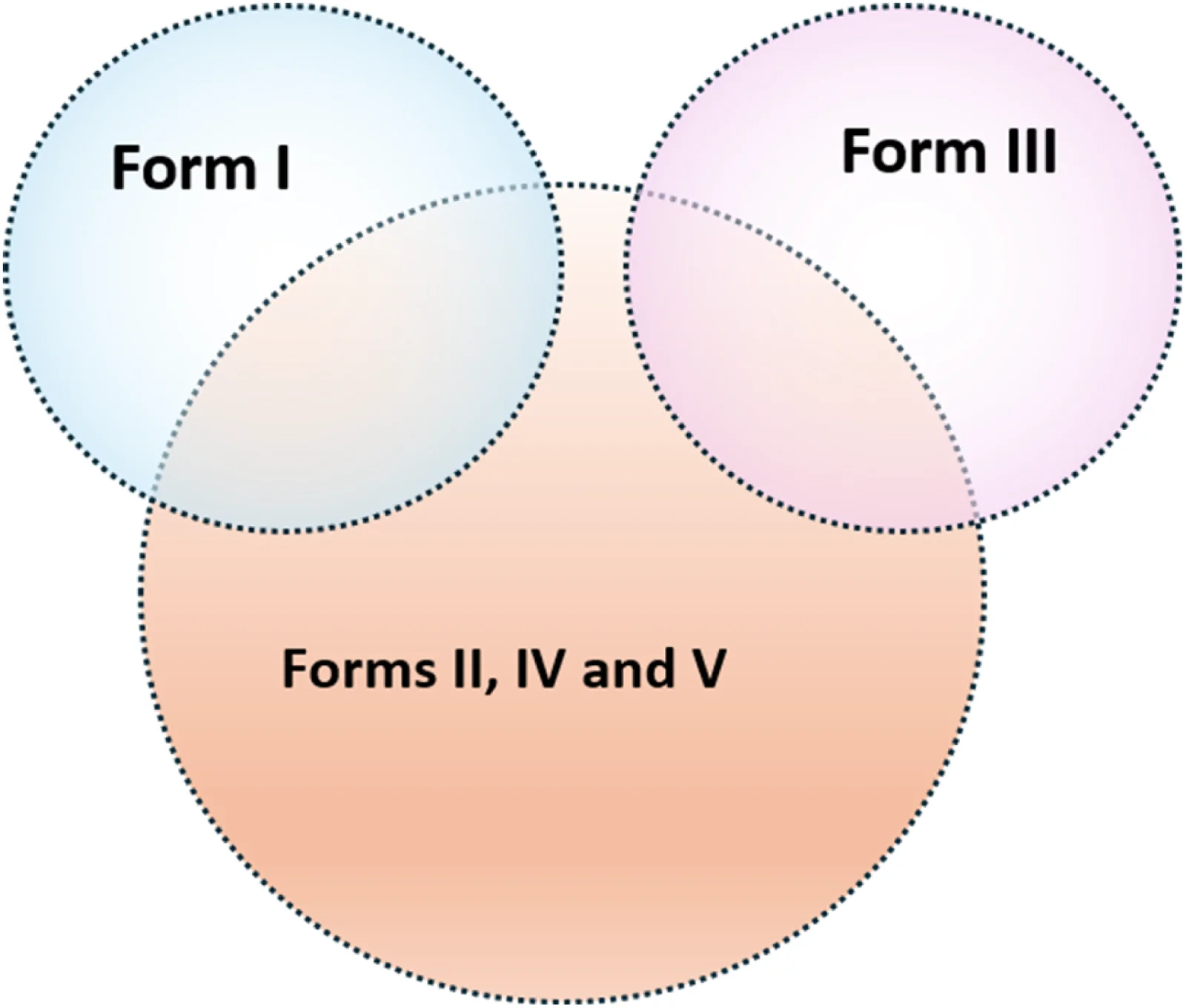
Venn diagram illustrating the conformational correlation among Forms I–V.

The variations in the observed torsion angles indicated that the PA molecule in each Form adopted different conformations, which may also account for the observed changes in the crystal packing among Forms I–V. Further, the pairwise molecular overlays indicated that while Forms I and III established conformational polymorphism with Forms II, IV, and V (RMSD range, 0.52–1.23 Å, > 0.345 Å), together, they represented only packing polymorphism, as overlay RMSD was only 0.262 Å (Table S2). The schematic is shown below (Scheme 2).

### Hirshfeld surface analysis

**Fig. 13:**
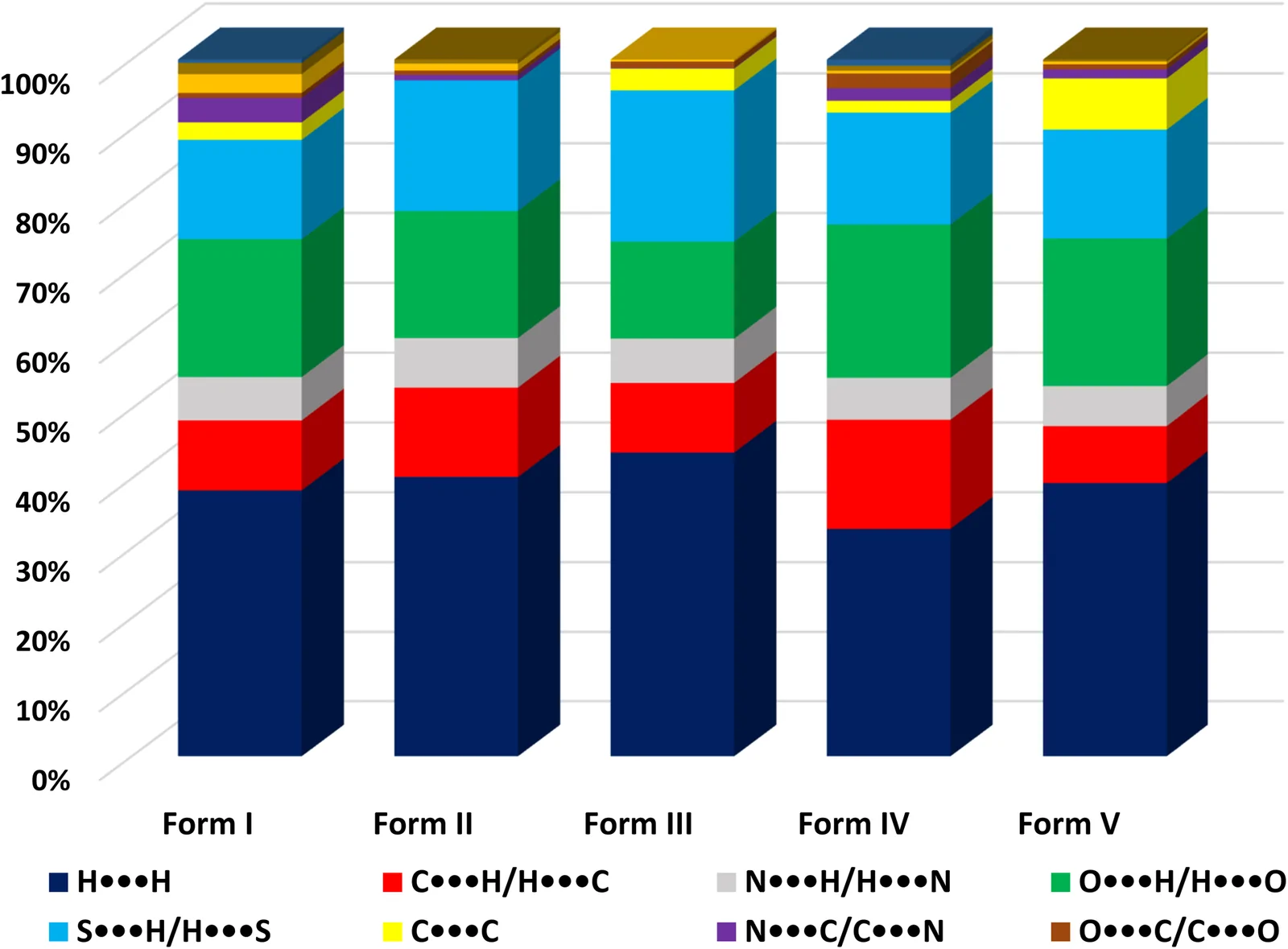
Relative contributions of different intermolecular interactions to the Hirshfeld surface area.

Hirshfeld surface analysis was performed to visualize and quantify the intermolecular interactions governing the crystal packing of the five polymorphs. The *d*_norm_ surfaces highlight the regions of close intermolecular contacts, while the two-dimensional fingerprint plots provide a quantitative description of the relative contributions of different interaction types based on the internal (*d*_i_) and external (*d*_e_) distances. The fingerprint plots for all five polymorphs are presented in Fig. S8, and the percentage contributions of the individual intermolecular contacts are summarized in Fig. 13.

It was clear that H⋯H contacts constituted the largest contribution to the Hirshfeld surface, ranging from 32.7% to 43.4%, reflecting the abundance of hydrogen atoms within the crystal structures. Also, since H⋯H contacts were predominantly dispersive and relatively weak, a relatively high proportion of these interactions suggested a low contribution from strong directional intermolecular interactions. Amongst the polymorphs reported herein, Form III exhibited the highest H⋯H contribution (43.4%), whereas Form IV showed the lowest value (32.7%).

The majority of red spots over the Hirshfeld surface were associated with the O⋯H/H⋯O, S⋯H/H⋯S and N⋯H/H⋯N contacts, contributing significantly to the stabilization of the crystal structures. The O⋯H/H⋯O interactions accounted for 13.8–21.9% of the Hirshfeld surface, with the highest contribution observed for Form IV (21.9%), followed closely by Form V (21.0%) Similarly, 5.7% to 7.1% of N⋯H/H⋯N interactions contributed to the Hirshfeld surface, with the largest contribution from Form III (7.1%) and the lowest contribution from Form V (5.7%). These percentages of interactions indicated that all polymorphs were primarily stabilized through a combination of strong polar hydrogen bonding interactions.

Furthermore, two other important interactions that contribute to the structural stabilization are π⋯π and C–H⋯π (with the former being more predominant). Here, the C–H⋯π interactions (C⋯H/H⋯C in the range of 8.1–15.6%) were predominant, with Form V exhibiting the lowest interaction, but the C⋯C (π ··· π) interactions were more effective contributors, as high as 7.3%, in Form V, while in all other structures, such contribution was almost negligible. Thus, Form V, though it had moderately high hydrophobic interactions, displayed a more balanced distribution of strong H-bonding interactions, together with complementary weak π⋯π and C–H⋯π contacts, consistent with its high packing efficiency and significant thermodynamic stability. Conversely, Form III was characterized by the largest proportion of weak H⋯H contacts (43.4%) and comparatively low contributions from strong hydrogen-bonding interactions (41.3%), which could be attributed to its low stability and perhaps the observed transformation of III → V.

### Lattice energy calculations

The relative thermodynamic stabilities of polymorphs I–V were evaluated by calculating their lattice energy (LE) using the Dmol3 module embedded in BIOVIA Materials Studio 2020. The calculated LE of different forms is listed in Table 5. Form V exhibited the lowest LE (−192.2 kJ mol^−1^), and Form III showed the highest (−185.4 kJ mol^−1^), with the stability order among the polymorphs being as follows: Form V > Form IV > Form II > Form I > Form III.

**Table 5:** Lattice energy of all forms of the PA and SA complexes

Forms	*E*_latt_ (kJ mol^−1^)
I	−186.9
II	−188.7
III	−185.4
IV	−190.2
V	−192.2

The high LE of Form III indicated that it had the lowest thermodynamic stability among the five polymorphs. Furthermore, the increasing order clearly corroborated the Hirshfeld analysis in terms of the contribution of different types of interactions towards the stabilization of the structures. Thus, Form III, which exhibited the highest contribution from H⋯H contacts, showed the highest LE, while Form V, with the lowest LE, was stabilized by directional H⋯O or N⋯O hydrogen-bonding interactions and π – π interactions. Thus, the computed LE was consistent with the experimentally observed solid-state transformation of Form III–V. However, the observed discrepancy between Δ*H* from DSC measurements and computed LE may be attributed to the significant difference in the crystalline environment and intermolecular interactions.^60^

### Solid-state UV-vis spectroscopy, photoluminescence and molecular orbital analysis

**Fig. 14:**
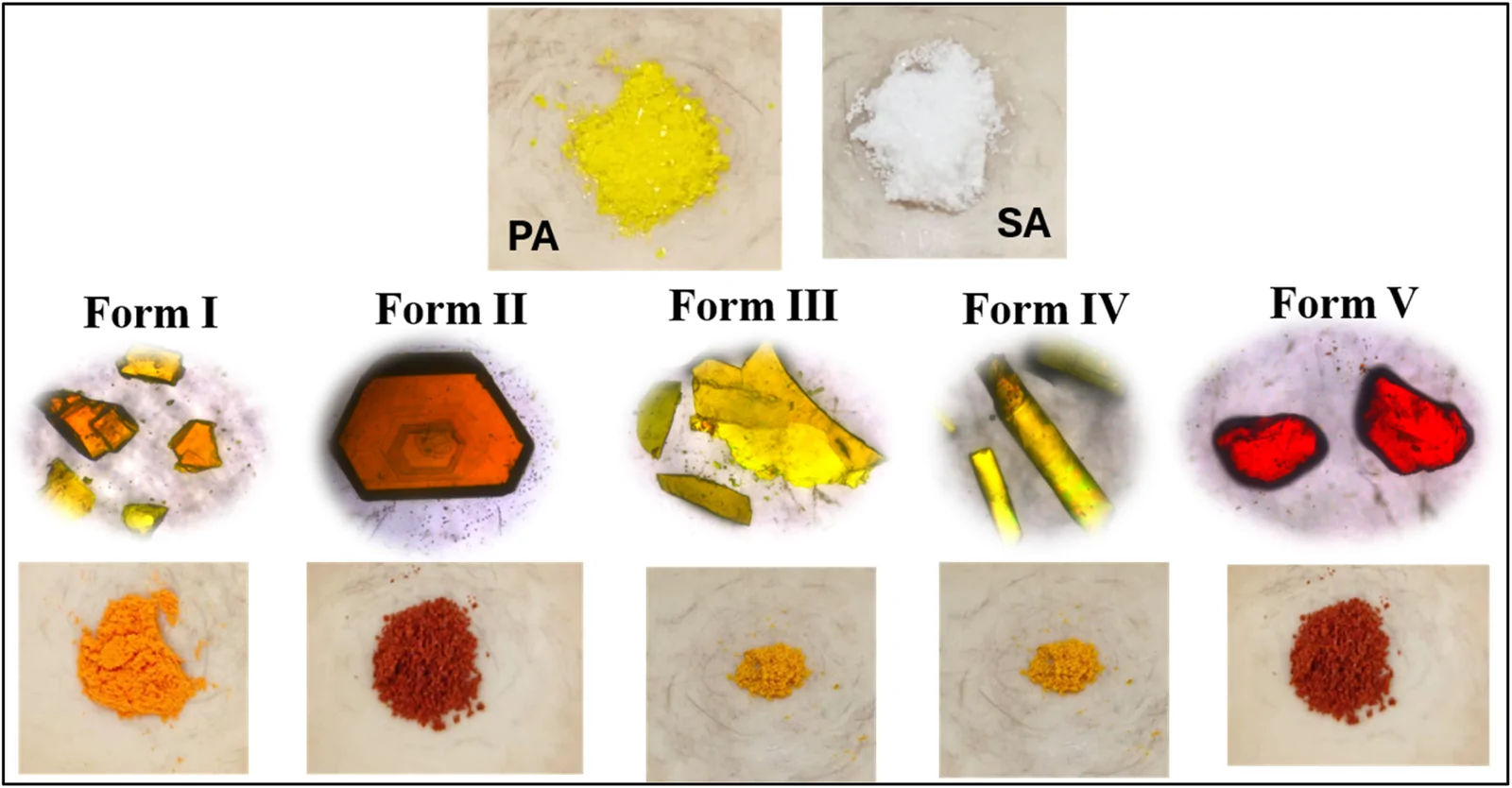
Colourful crystals and the corresponding powdered samples of the forms (I–V).

A fascinating feature of the co-crystal polymorphs, I–V, was the appearance in distinct colours (yellow, orange, and red) despite having the same chemical composition (Fig. 14). In general, as the appearance of a colour was due to electronic transitions, the observed intriguing colour polymorphism in the crystals of I–V was investigated by solid-state UV-vis diffuse reflectance spectroscopy, photoluminescence spectroscopy, and DFT-based molecular orbital calculations. These complementary techniques provided experimental and theoretical evidence for the relationship between crystal packing and the observed optical behaviour.

**Fig. 15:**
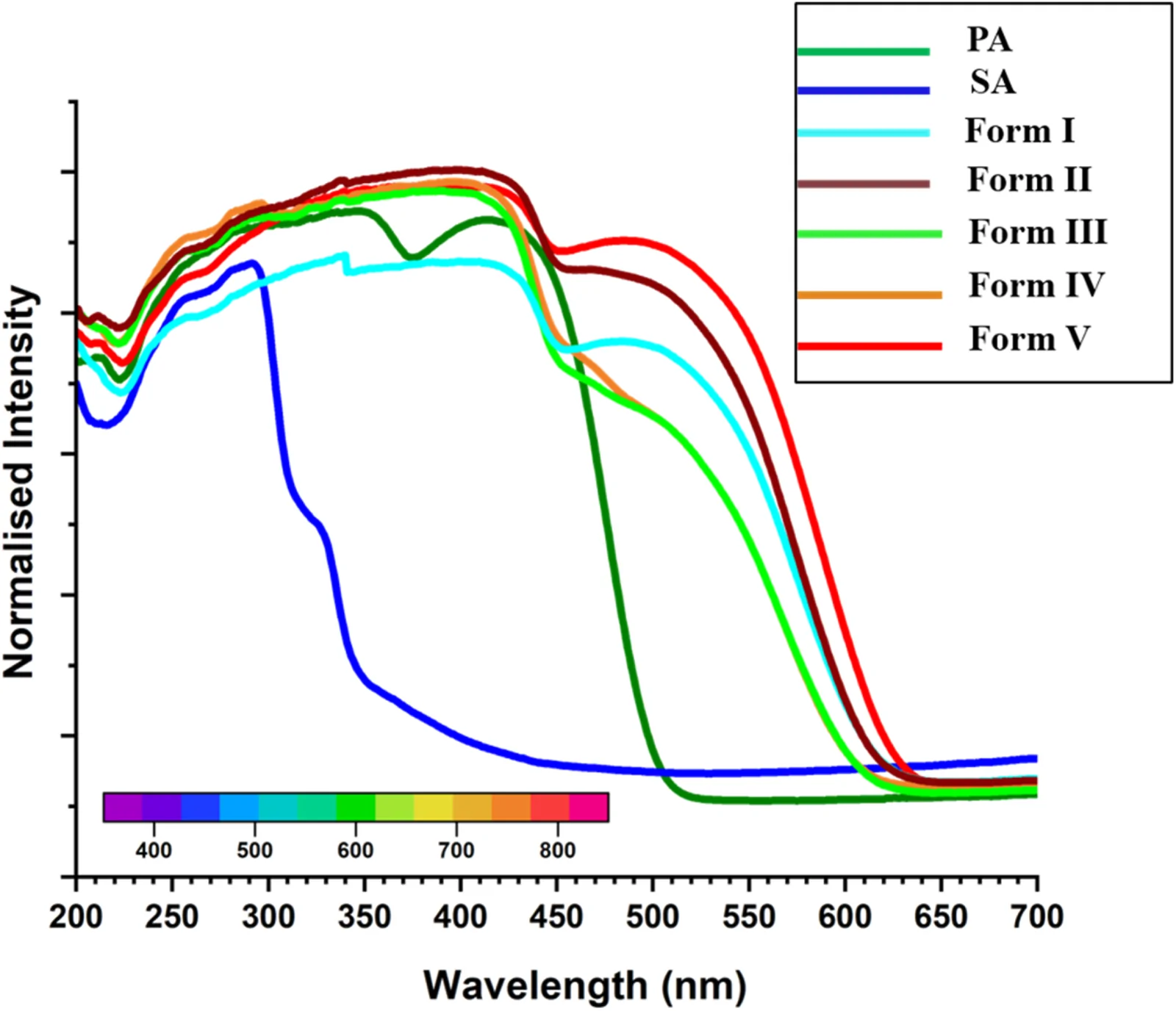
Solid-state UV-vis plots of PA, SA and all the forms (I–V) of PA–SA salts.

The normalized diffuse reflectance spectra of PA, SA, and Forms I–V are shown in Fig. 15. While pristine PA exhibited an absorption edge near 508 nm, polymorphs I–V displayed absorption extending further into the visible region, confirming that co-crystallization substantially modified the electronic transitions. Among the polymorphs, Forms V and II exhibited the most pronounced bathochromic shift, with an absorption edge extending to approximately 630 nm, whereas Forms III and IV showed comparatively shorter absorption edges. The trend in the absorption edge followed Form V (630 nm) > Form II (628 nm) > Form I (627 nm) > Form III (618 nm) ~ Form IV (618 nm) > PA (508 nm), consistent with the progressive decrease in the observed optical transition energy.

**Fig. 16:**
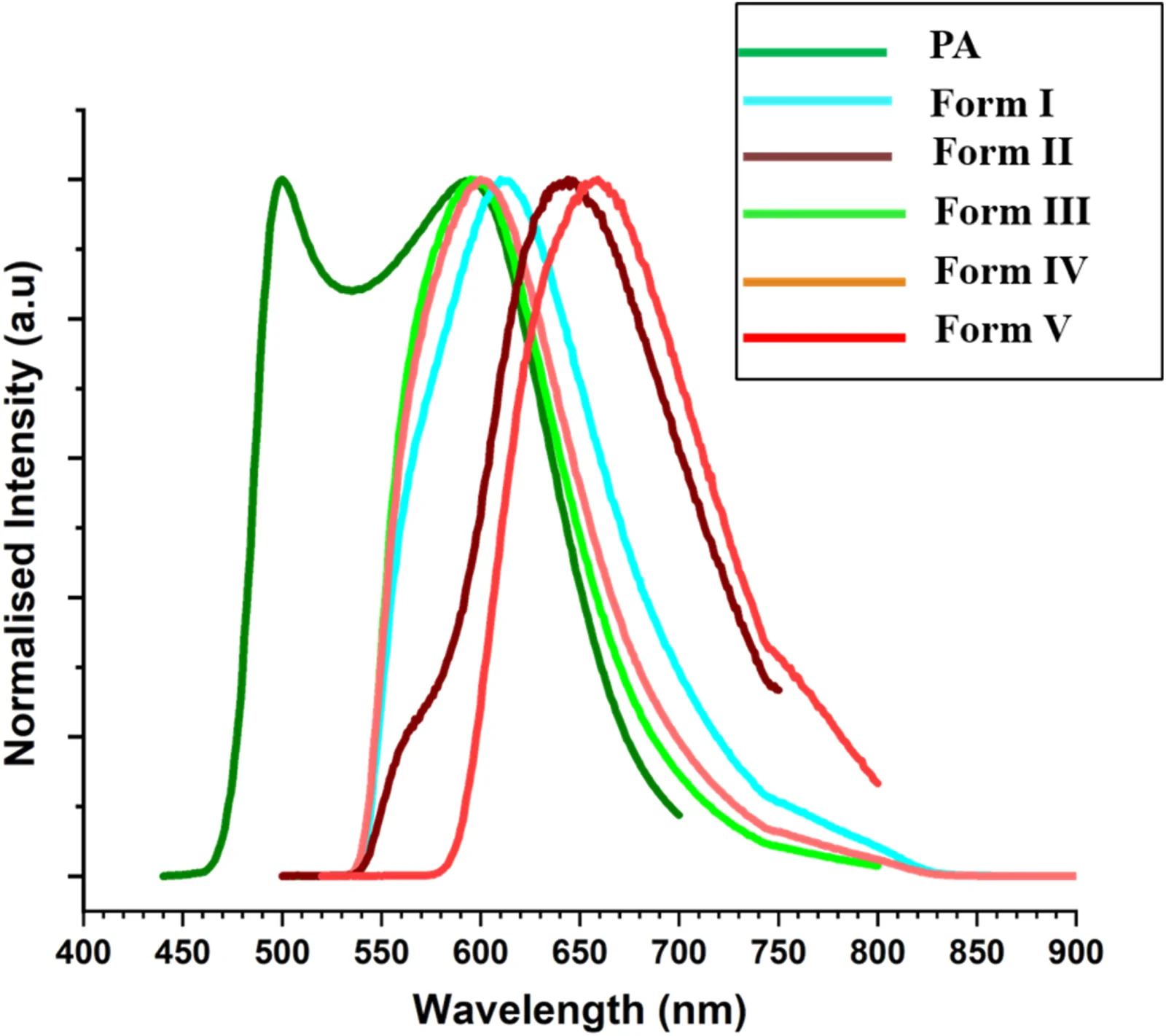
Solid-state emission spectra of PA and all the forms (I–V) of the PA–SA complex.

To further evaluate the electronic origin of the optical properties, molecular orbital calculations were performed on the structures of I–V. The molecular orbital diagrams of all the forms and PA, with the corresponding energy gaps, are shown in Fig. S9. The HOMO was predominantly localized over the SA fragment, whereas the LUMO was mainly distributed on the PA fragment, indicating that the lowest-energy electronic excitation showed a significant intermolecular charge-transfer character. Such a donor–acceptor orbital separation was facilitated by the effective molecular recognition between the PA and SA molecules in the co-crystals through strong hydrogen bonding. Variations in the crystal packing among the polymorphs modulated the extent of orbital overlap and intermolecular electronic coupling, leading to progressive changes in the HOMO–LUMO energy gap. The calculated decrease in the energy gap from 2.156 eV (Form III) to 1.661 eV (Form II) correlated well with the experimentally observed bathochromic shift in the diffuse reflectance, providing a molecular-level explanation for the yellow to red color polymorphism. These calculations were further authenticated by the spectroscopic measurements through photoluminescence emission spectra (Fig. 16).

The polymorphs exhibited relatively small HOMO–LUMO gaps, corresponding to red shifts (emission at a long wavelength) in the emission maxima of PA (592 nm) → Forms I–V (600–665 nm), which were in agreement with the diffuse reflectance measurements. These observations confirmed that variations in the crystal packing altered both the ground-state and excited-state electronic structures, giving rise to the distinct optical properties of Forms I–V.

## Conclusions

This study elucidated one of the most extensive polymorphic landscapes reported for a binary pharmaceutical co-crystal, established through the systematic screening of crystallization solvents and temperature. The identification of five distinct forms of the PA and SA system through a conventional slow evaporation method served as a definitive model for the role of conformational flexibility in directing supramolecular assembly. All polymorphs exhibited vibrant colours with distinct crystal habits.

**Scheme 3:**
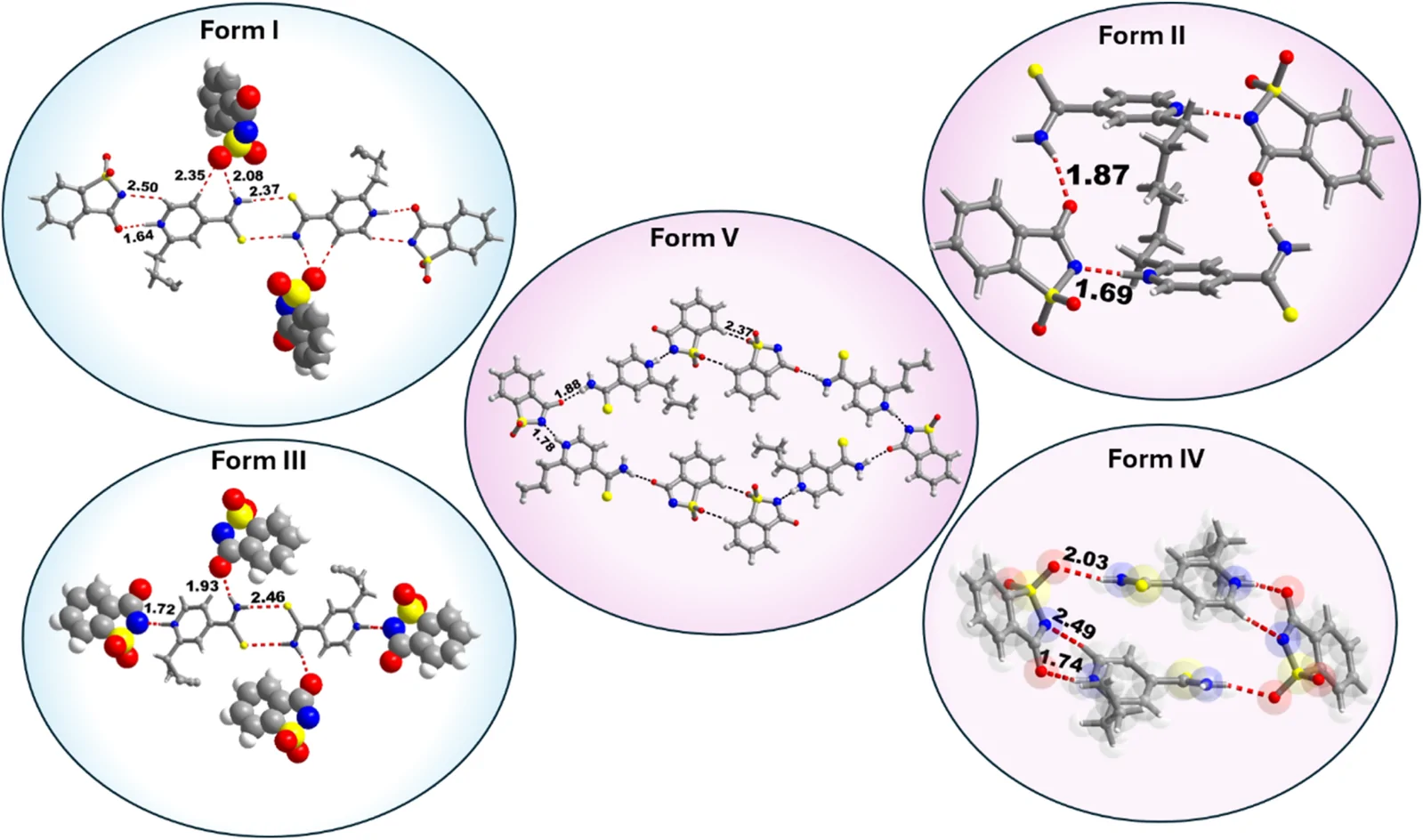
Hydrogen bonding in Forms I-V.

Structural analysis revealed that the diverse hydrogen-bonding topologies, ranging from homomeric dimers to complex heteromeric quaternary cycles, were driven by the flexibility of the thioamide (–CSNH_2_) and propyl (–C_3_H_7_) substituents of PA, as shown in Scheme 3. Further, Forms I, II, IV, and V exhibited high thermodynamic stability, excluding Form III.

This study provides a possible roadmap for understanding the structural hierarchy in co-crystals. Furthermore, the endeavors offer critical insights to enhance the exploration of polymorphism, particularly for multi-component active pharmaceutical ingredients (APIs), to navigate the polymorphic risks, as many API-based co-crystals have been developed in recent times.

## Conflicts of interest

The authors declare no conflicts of interest.

## Note added after first publication

This article replaces the version published on 25th August 2026, in which the structure image from Table 4 had been replaced with the image now included as Scheme 3 in the conclusions.

## Supplementary Material

RA-016-D6RA03965K-s001

RA-016-D6RA03965K-s002

## Data Availability

CCDC 2292144–2292148 contain the supplementary crystallographic data for this paper.^61^ The data can be obtained free of charge from the Cambridge Crystallographic Data Centre via https://www.ccdc.cam.ac.uk/structures/. Supplementary information (SI): additional crystallographic, experimental, and computational data supporting the characterization of Forms I–V, including PXRD, DSC, Hirshfeld surface analysis, RMSD, HOMO–LUMO analysis, and thermodynamic calculations. See DOI: https://doi.org/10.1039/d6ra03965k.
