# Supplementary material for: Co-crystal polymorphism: five colourful forms of prothionamide-saccharin co-crystal
Source: RSC Adv. 2026 Aug 25;16(42):50358–73. doi: 10.1039/d6ra03965k (PMC13505303; doi:10.1039/d6ra03965k)
Supplement: Supplementary file 1 [file RA-016-D6RA03965K-s001.pdf]

Supplementary Information (SI) for RSC Advances.
This journal is © The Royal Society of Chemistry 2026

Supporting Information (SI)

Co-crystal Polymorphism: Five Colourful Forms of Prothionamide-Saccharin Co-crystal

Sonali Ghosh*, Samina Easmin, and Venkateswara Rao Pedireddi

Solid State & Supramolecular Structural Chemistry Laboratory, Department of Chemistry, School of Basic Sciences, Indian Institute of Technology Bhubaneswar, Argul, Bhubaneswar 752 050, India.

Email: sg46@iitbbs.ac.in

Table of Contents.	Page No.
1. ORTEP diagram of Forms **II** - **V**	3 - 4
2. Comparative PXRD patterns (experimental and simulated) of Forms **I** - **V**	5 - 7
3. DSC plots showing heating and cooling cycles	8 - 10
3. Crystallization trial	11
4. Pairwise RMSD and maximum atomic displacement (Max. D) values among the Forms **I** - **V**	12
5. Hirshfeld surface and corresponding fingerprint plot of all Forms	13 -14
6. HOMO and LUMO energy diagram	15
7. Thermodynamics calculation for the transformation Form **III** → Form **V**	16 - 17

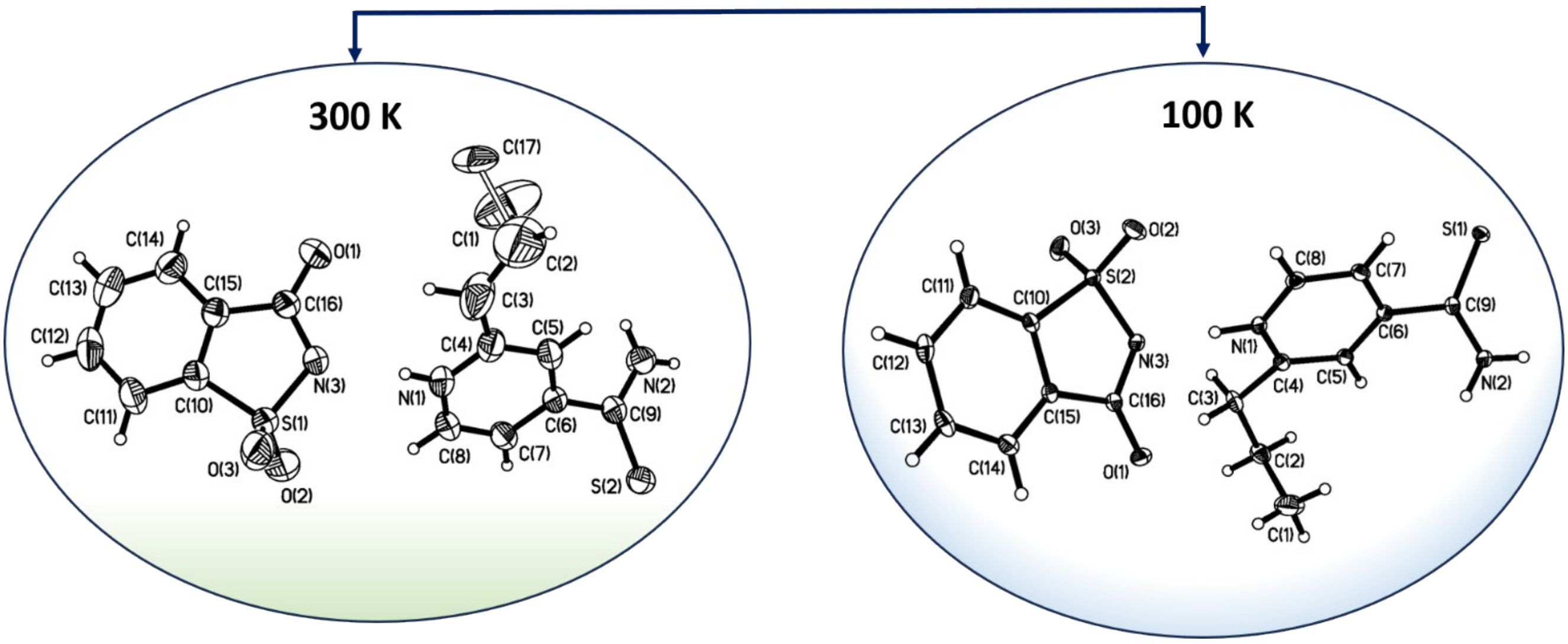

Figure S1. ORTEP representation of the asymmetric unit in Form **II** (a) 300 K and (b) 100 K.

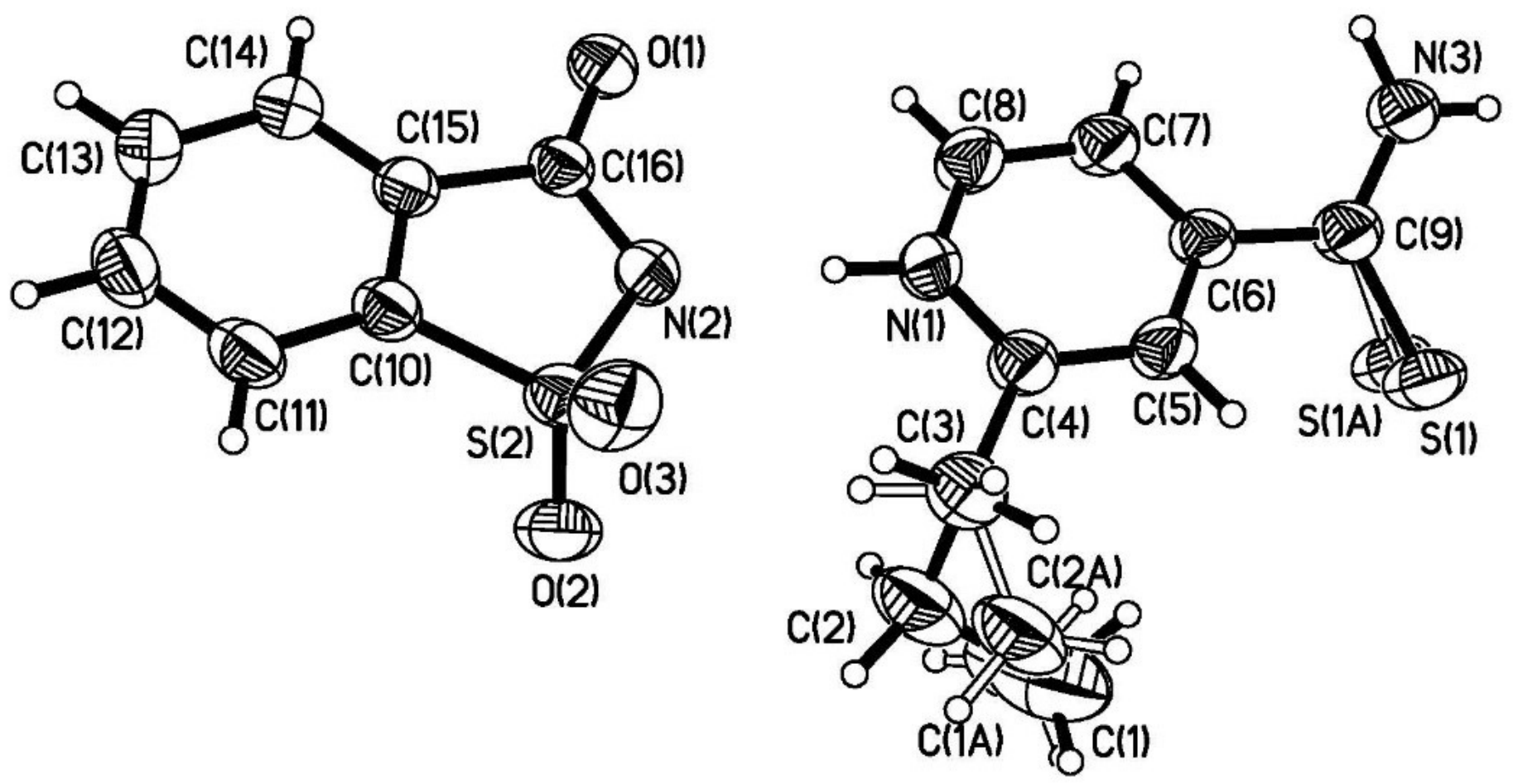

Figure S2. ORTEP of the contents of asymmetric unit in Form **III**.

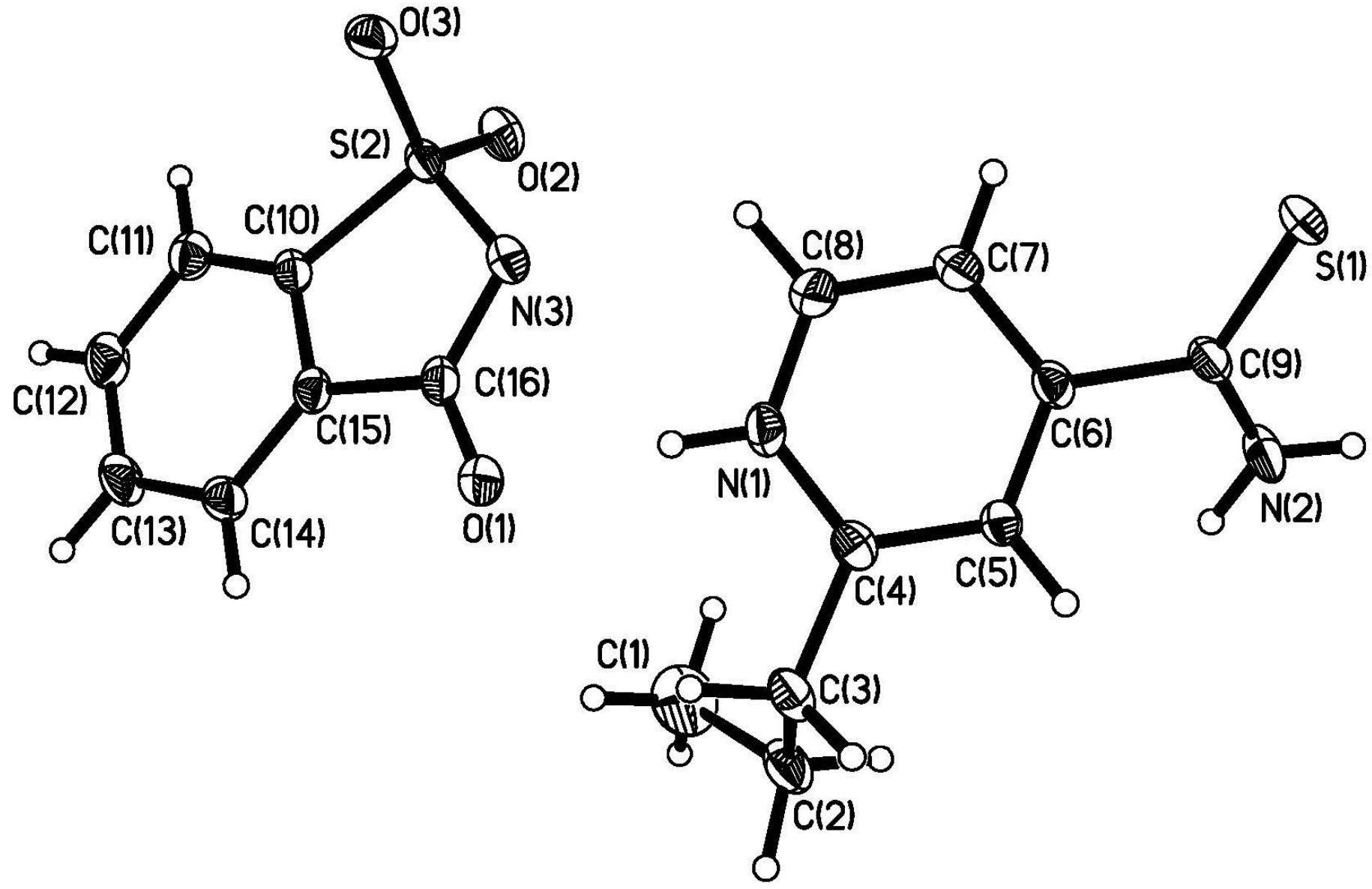

Figure S3. Asymmetric unit of Form **IV** represented in the form of ORTEP diagram.

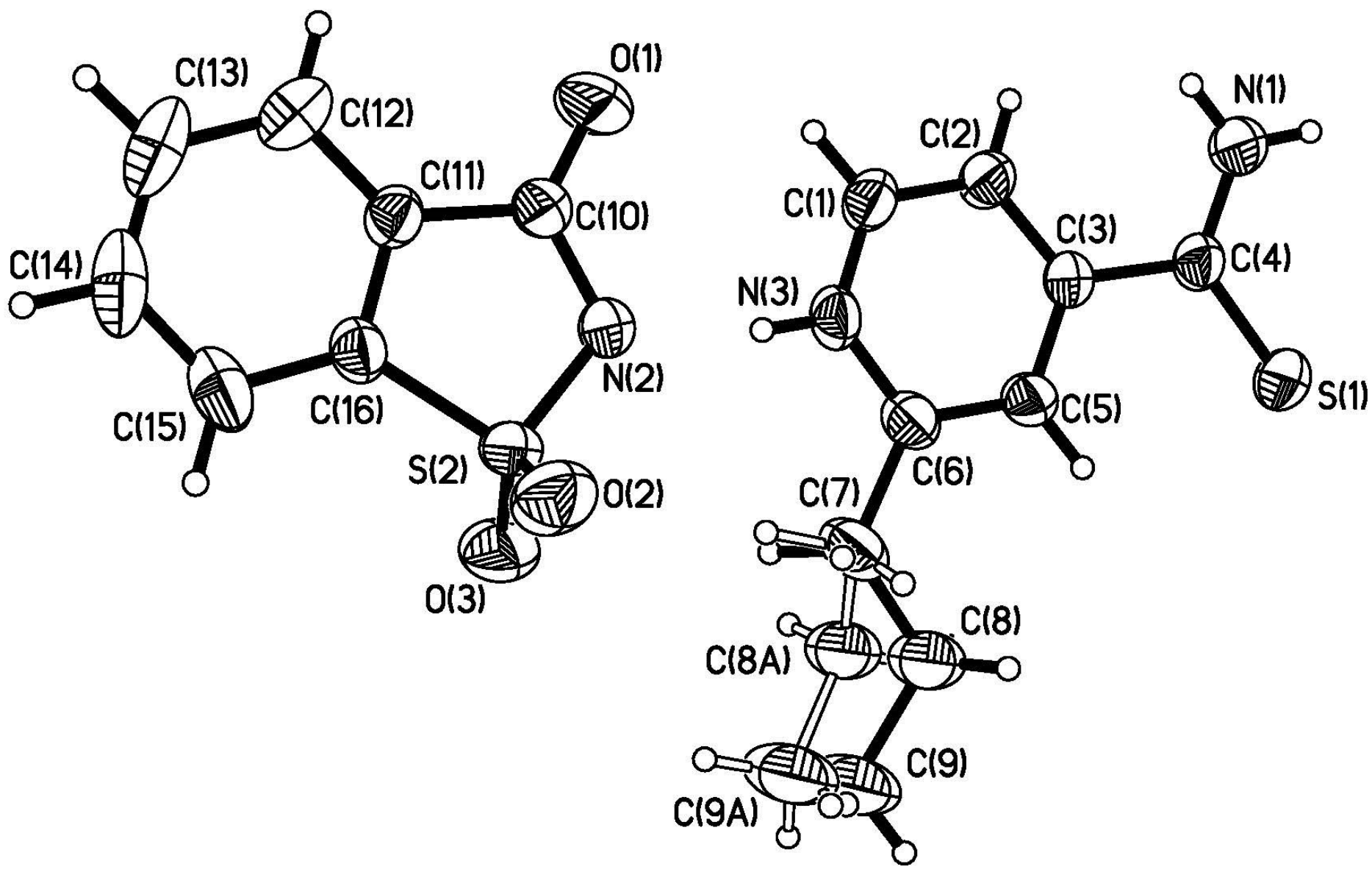

Figure S4. ORTEP projection of the asymmetric unit in Form **V**.

PXRD Studies.

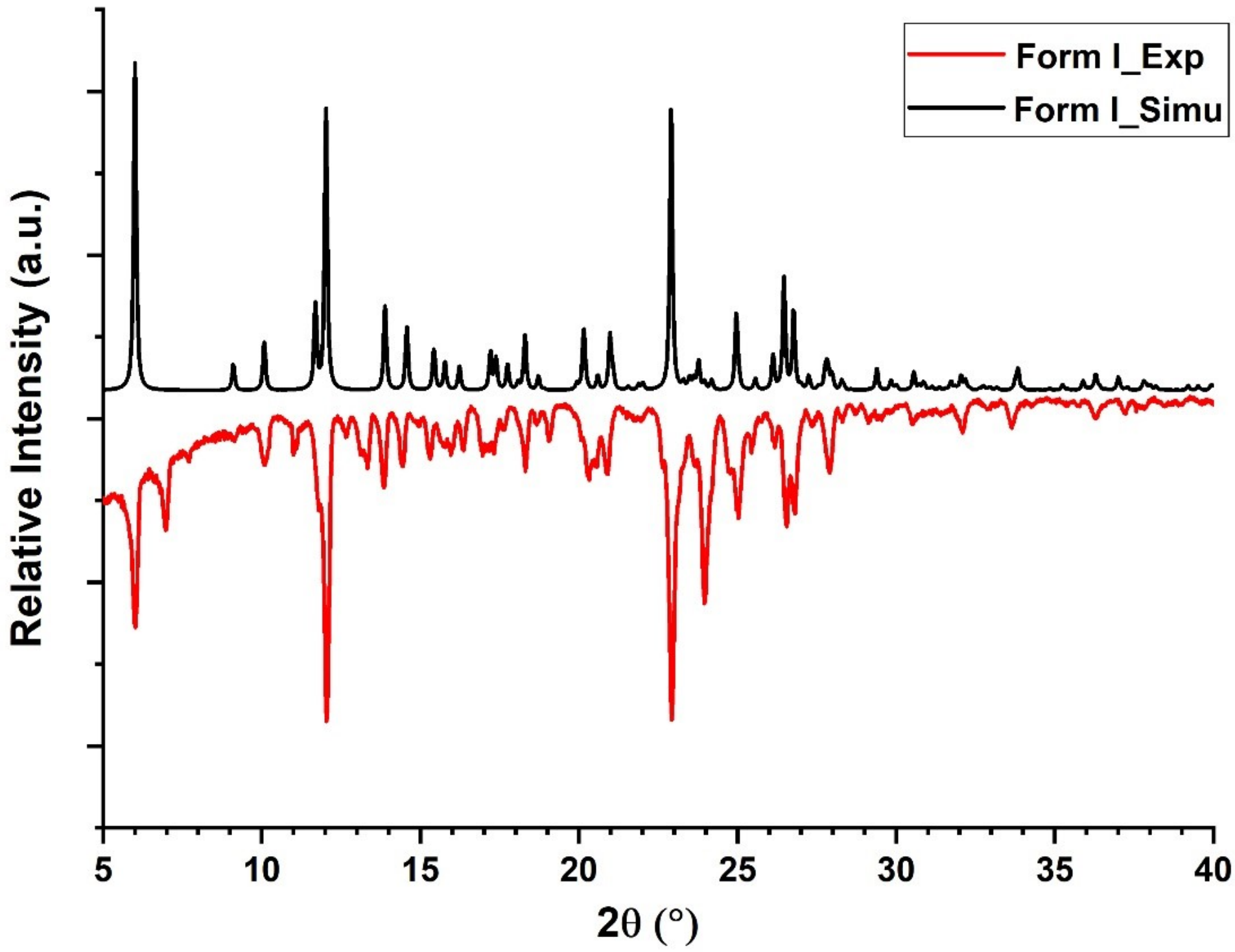
Form I_Exp
Form I_Simu
Relative Intensity (a.u.)
5
10
15
20
25
30
35
40
2θ (°)

(a)

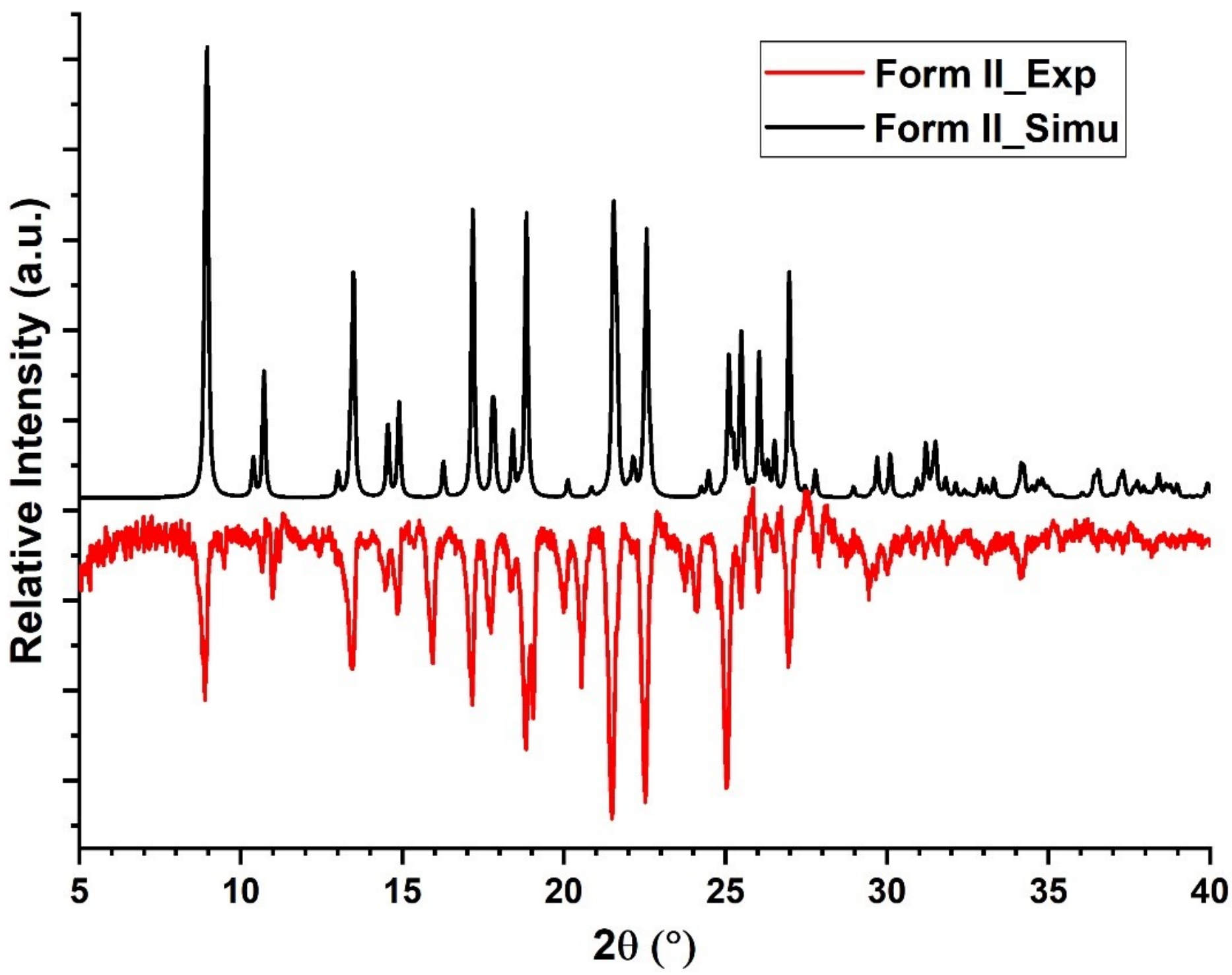
Form II_Exp
Form II_Simu
Relative Intensity (a.u.)
5
10
15
20
25
30
35
40
2θ (°)

(b)

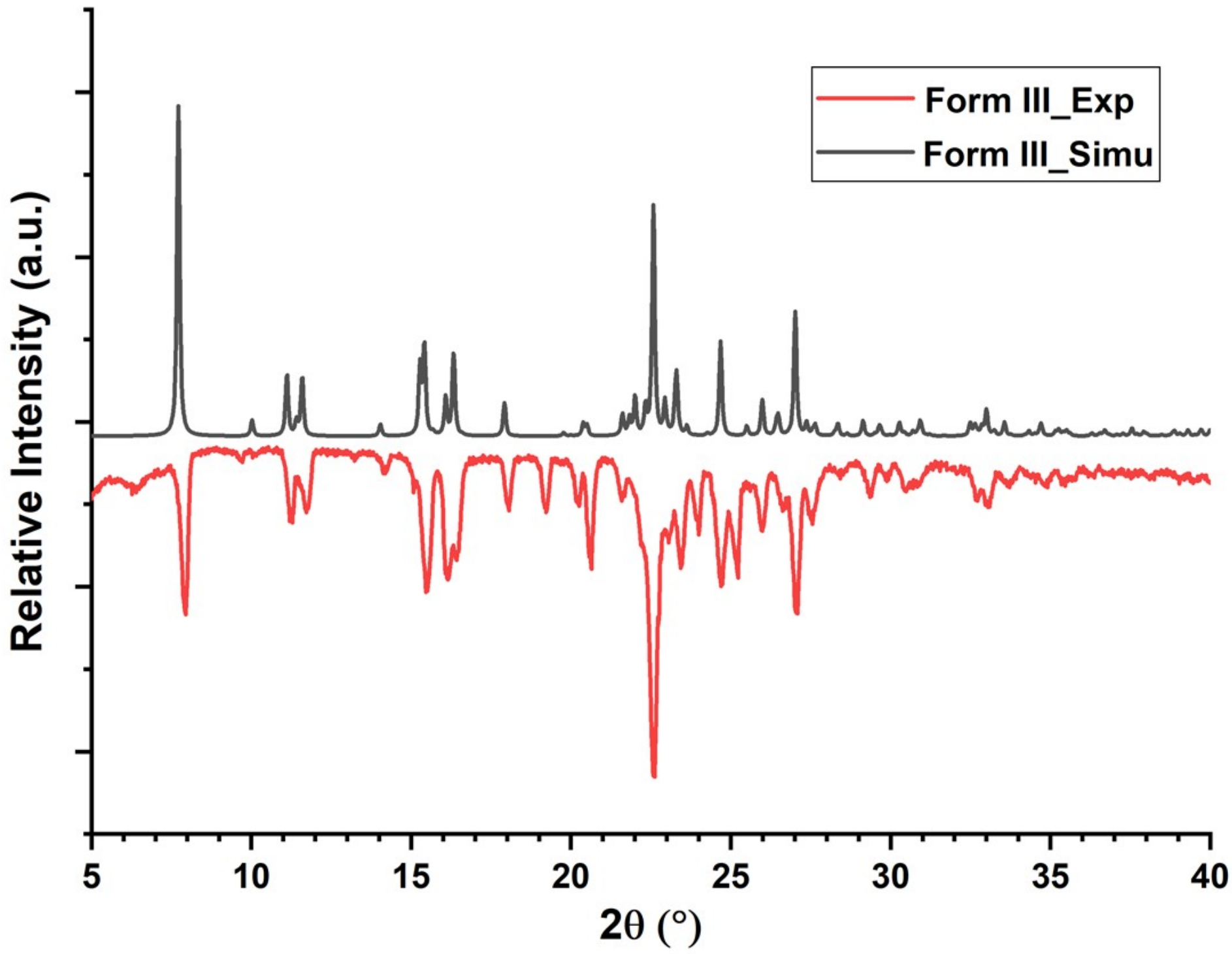

Form III_Exp
Form III_Simu
Relative Intensity (a.u.)
5
10
15
20
25
30
35
40
2θ (°)

(c)

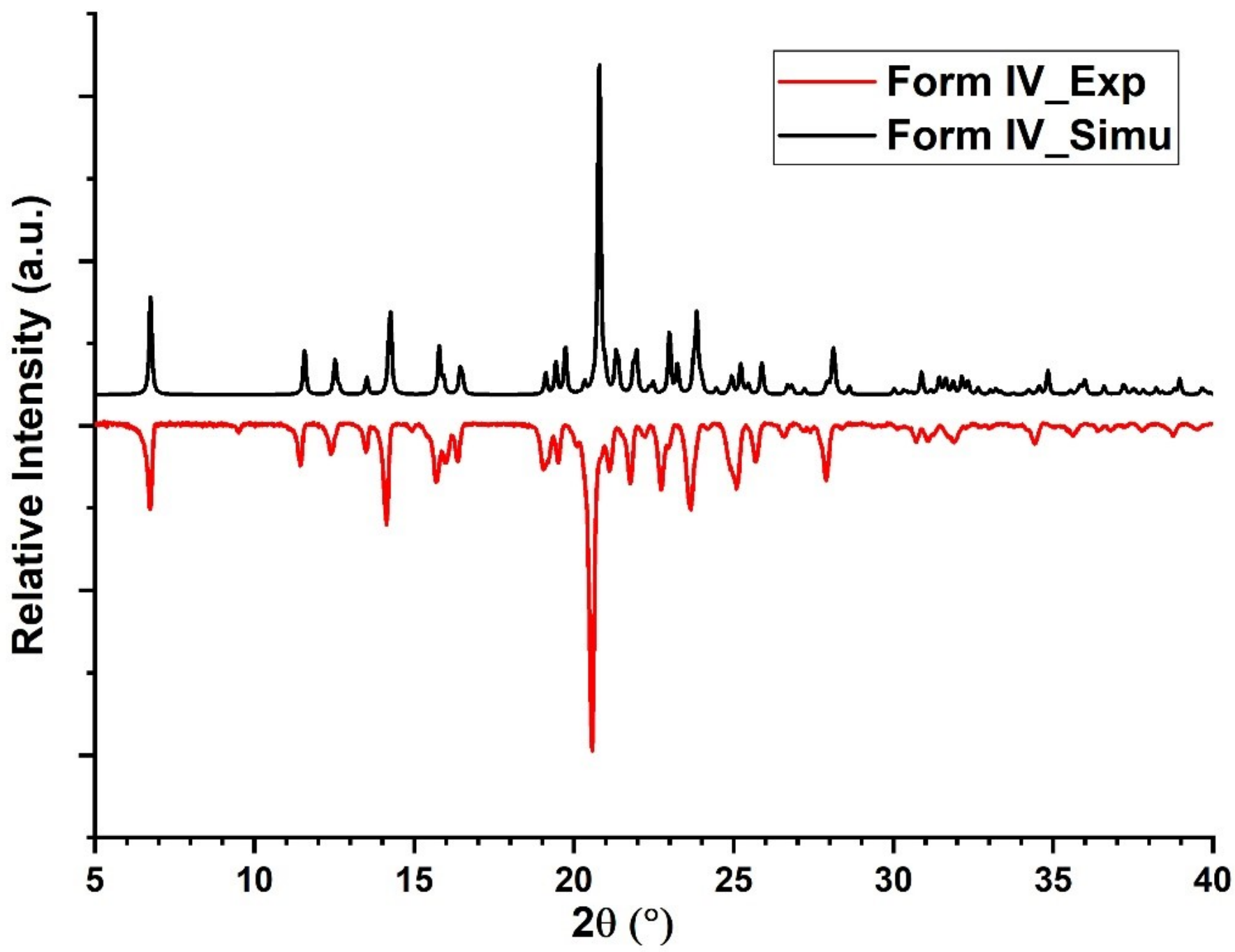

Form IV_Exp
Form IV_Simu
Relative Intensity (a.u.)
5
10
15
20
25
30
35
40
2θ (°)

(d)

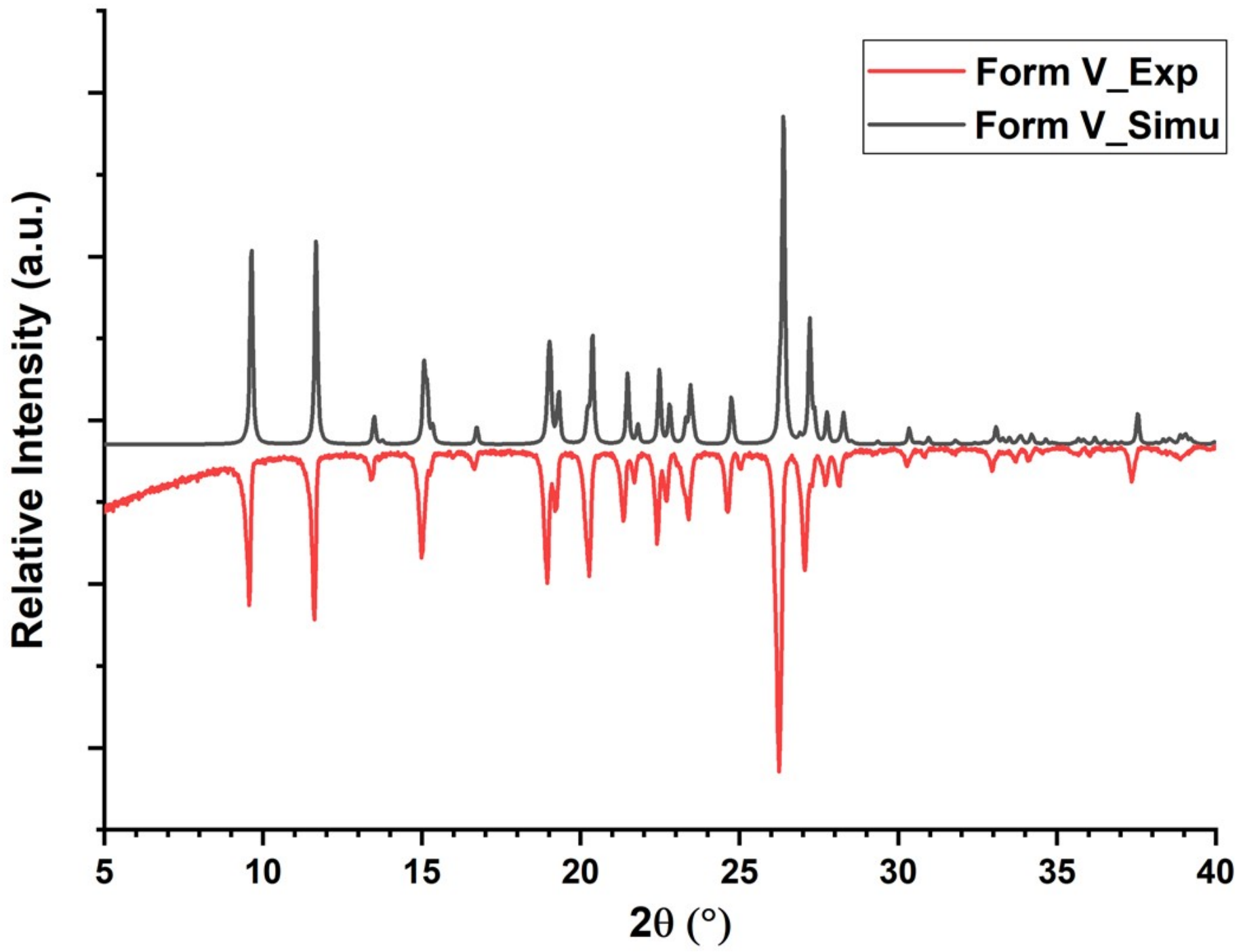

(e)

Figure S5. Experimental and simulated PXRD patterns of (a) Form **I**, (b) Form **II**, (c) Form **III**, (d) Form **IV**, and (e) Form **V**.

DSC Plots.

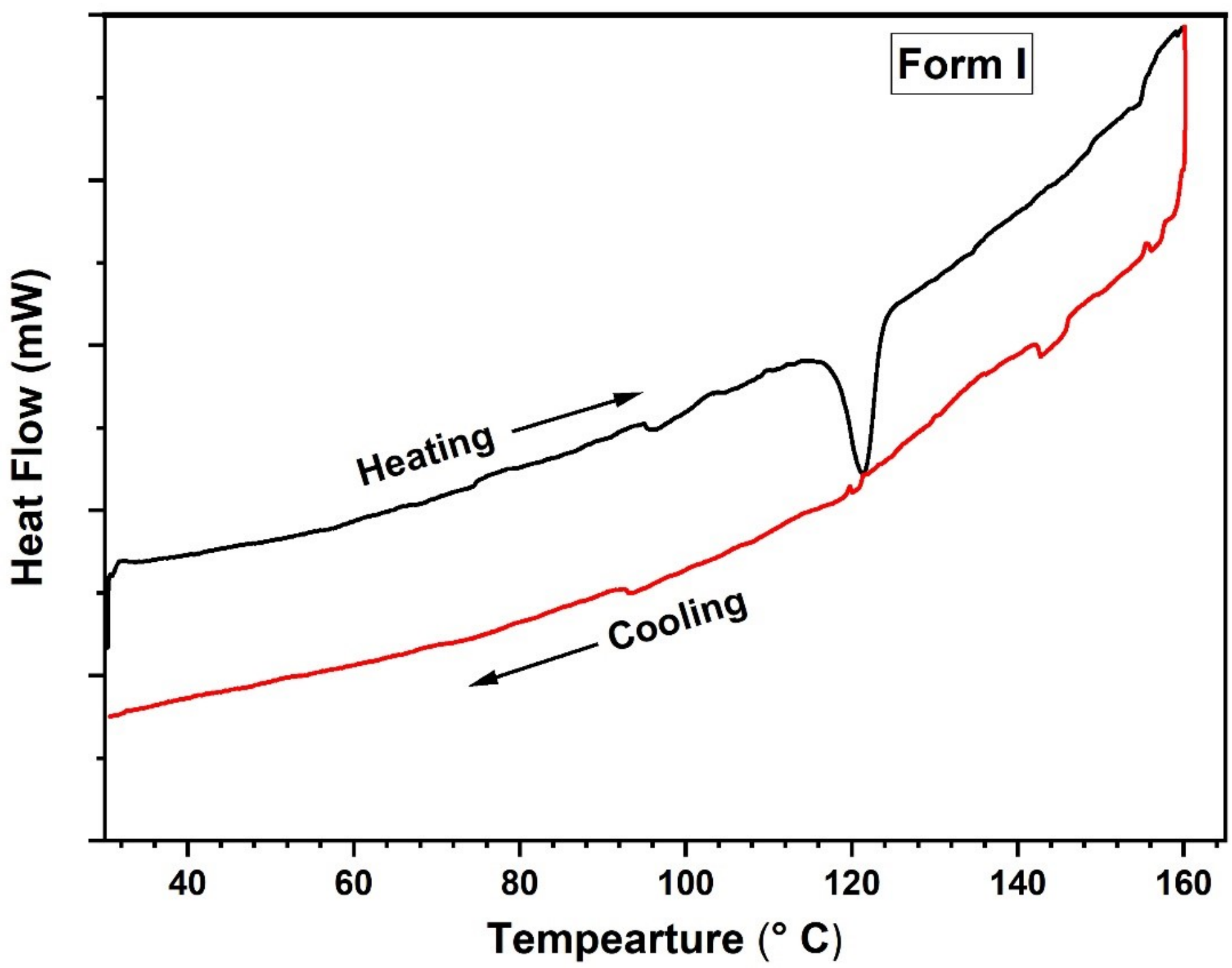

(a)

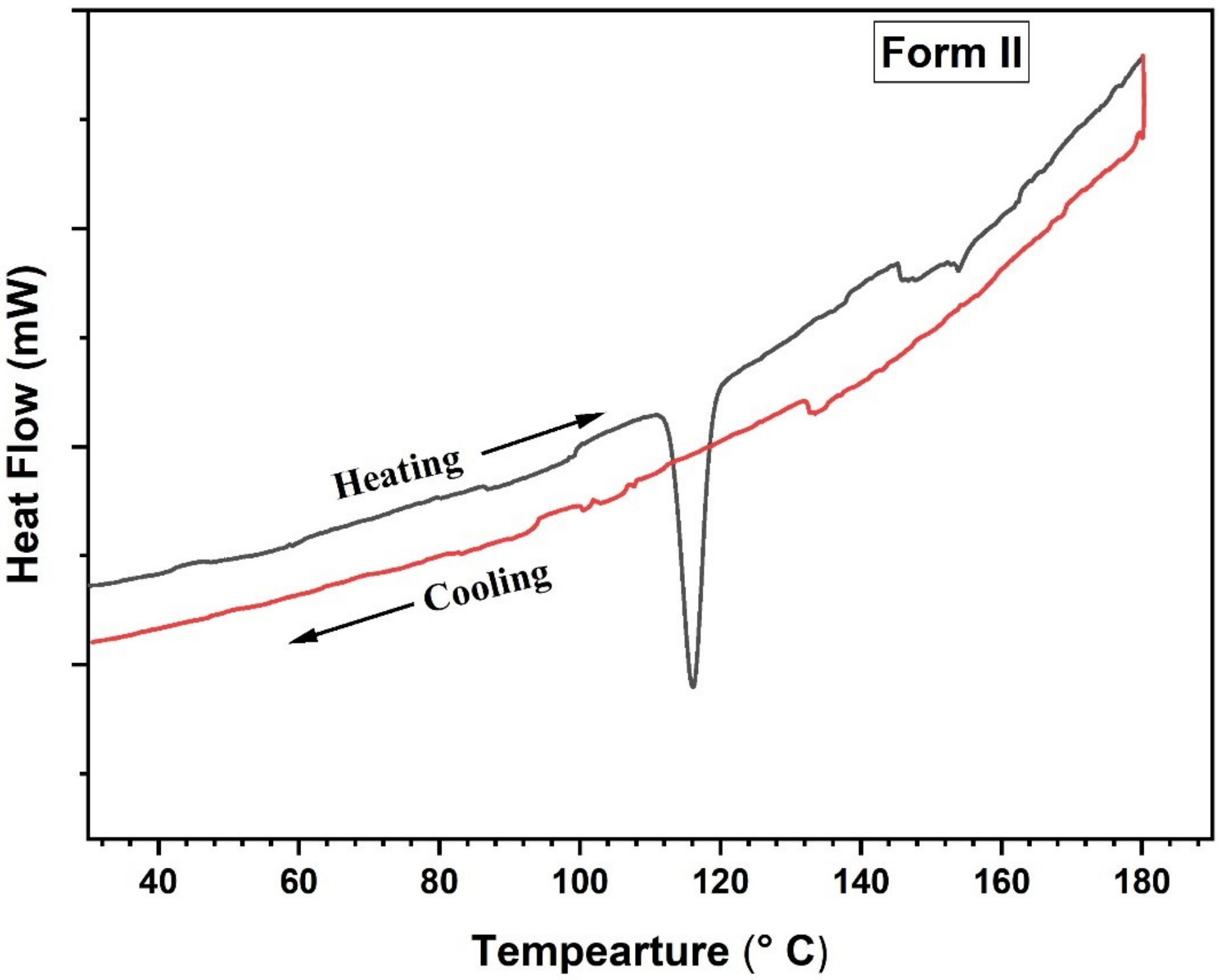
Form II
Heating
Cooling
Heat Flow (mW)
40
60
80
100
120
140
160
180
Tempearture (° C)

(b)

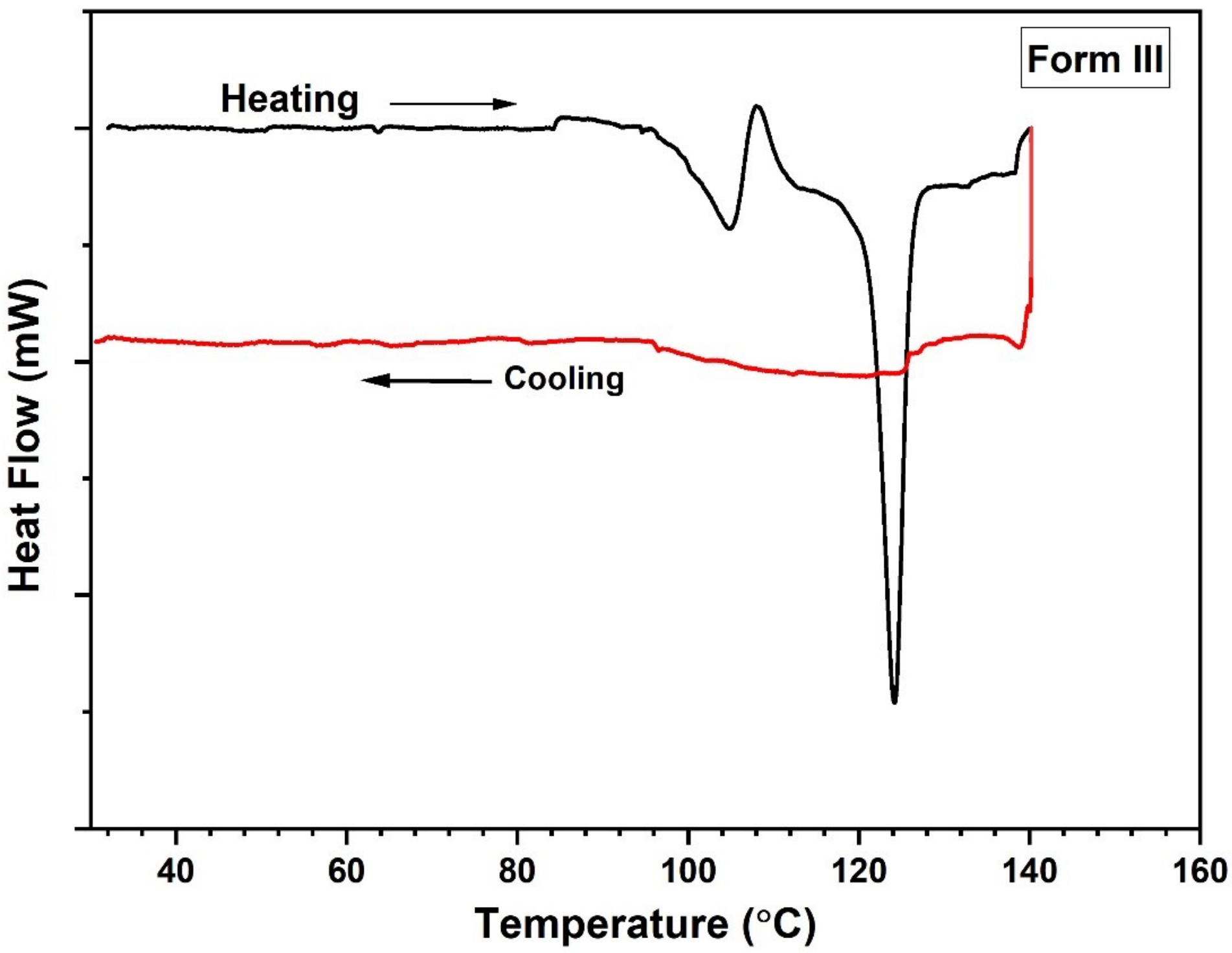
Form III
Heating
Cooling
Heat Flow (mW)
40
60
80
100
120
140
160
Temperature (°C)

(c)

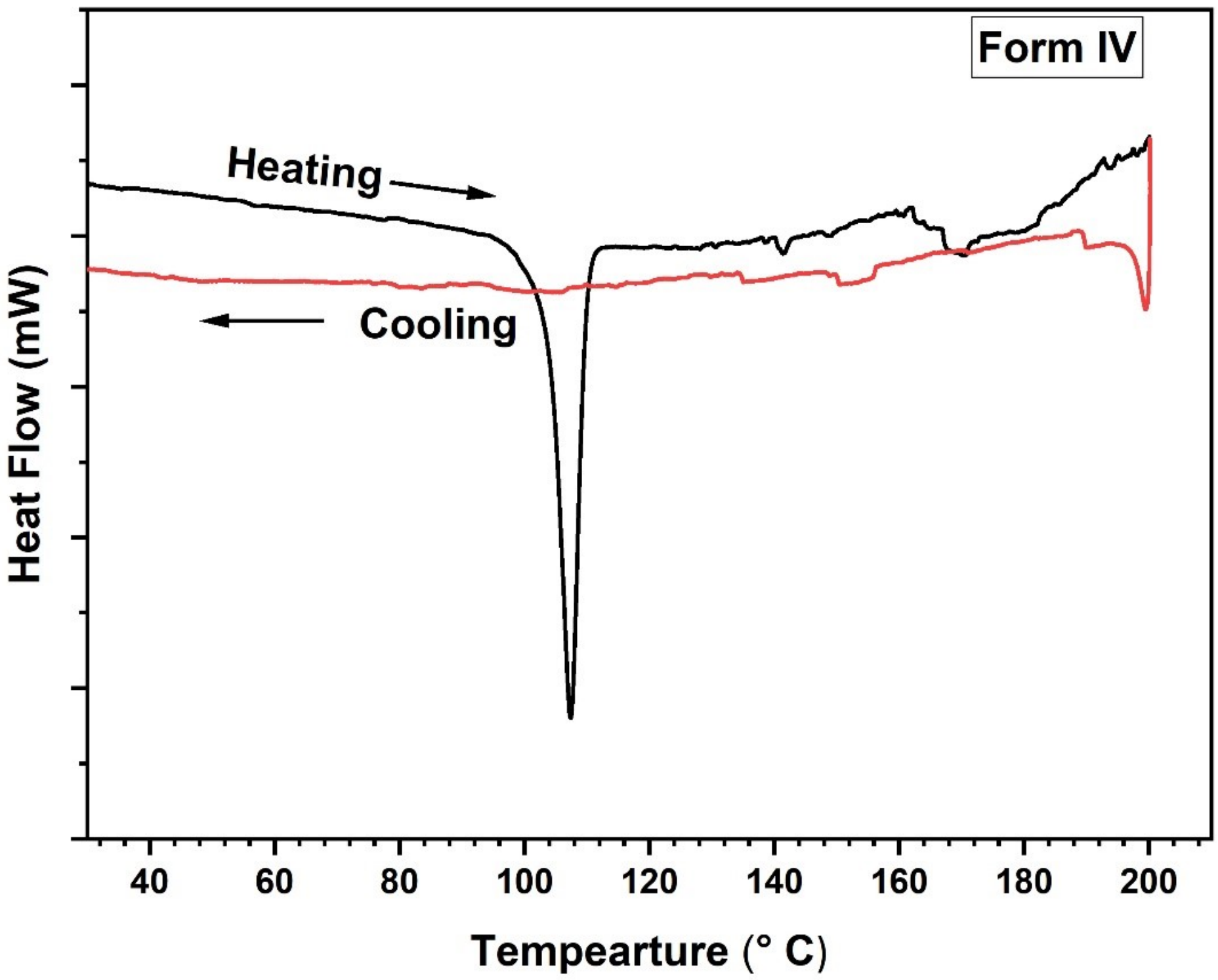

Form IV
Heating
Cooling
Heat Flow (mW)
40
60
80
100
120
140
160
180
200
Tempearture (° C)

(d)

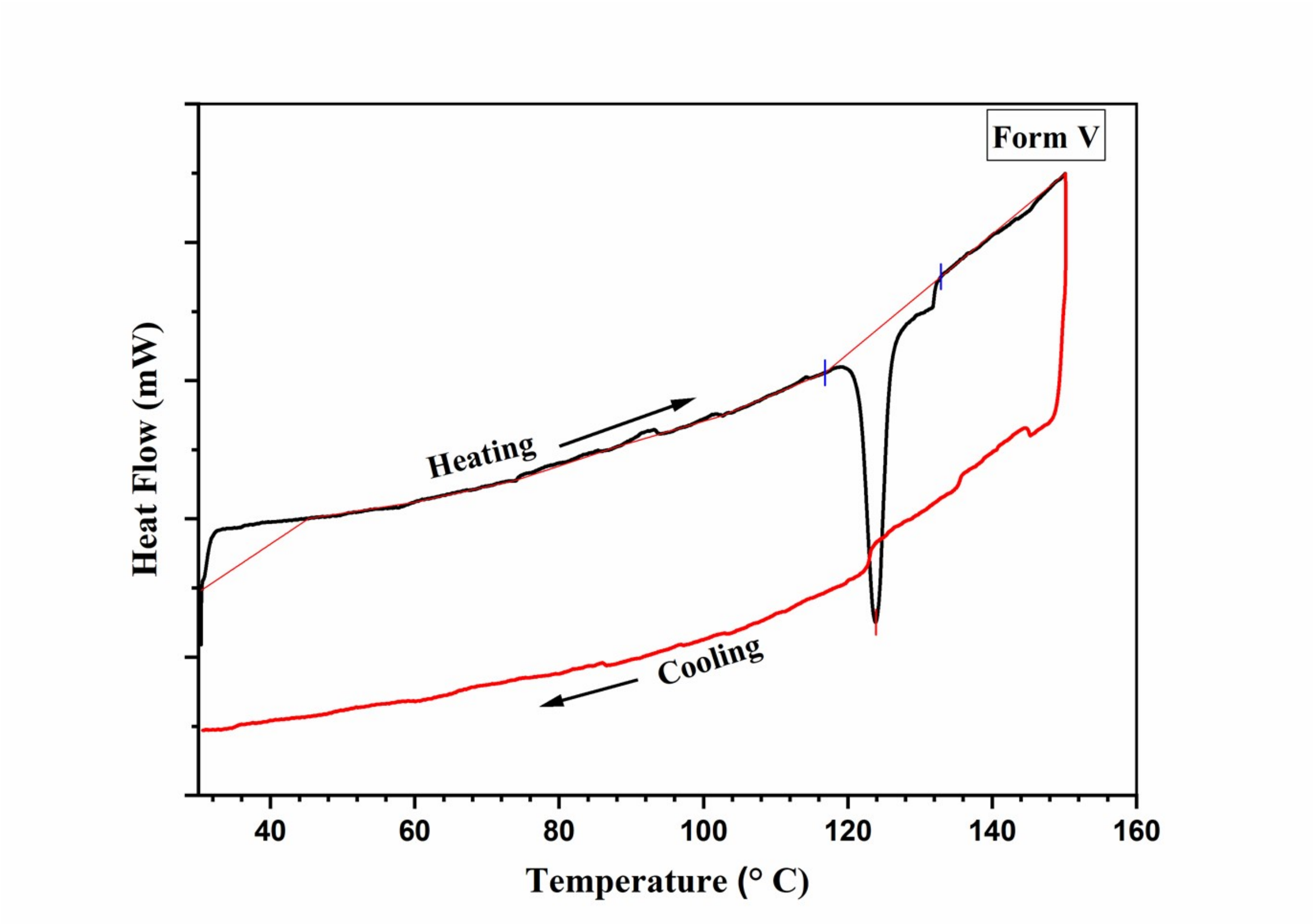

(e)

Figure S6. DSC plots showing heating and cooling cycles in (a) Form **I**, (b) Form **II**, (c) Form **III**, (d) Form **IV** and (c) Form **V**.

Table S1. Crystallization trial with different solvent and mixture of solvents.

	Solvents	I	II	III	IV	V

		Forms				
1.	EtOAc (RT)	⚐				
2.	EtOAc (LT)		⚐			
3.	EtOAc + MeOH (2:1)			⚐		
4.	EtOAc + CH_3CN (2:1)				⚐	
5.	EtOAc + n-hexane (2:1)					⚐
6.	CH_3COCH_3					⚐
7.	DCM					⚐
8.	Ethylene-1,2-dichloride					⚐
9.	DCM + n-hexane (2:1)					⚐
10.	Ethylene-1,2-dichloride + n-hexane (2:1)					⚐
11.	CH_3COCH_3 + n-hexane (2:1)					⚐
12.	EtOH + n-hexane (2:1)					⚐
13.	Melt Crystallization					⚐
14.	MeOH	No crystals				
15.	EtOH					
16.	CH3CN					
17.	CH_3NO_2					
18.	THF					
19.	1,2-Dioxane					
20.	MeOH + n-hexane (2:1)					
21.	CH_3CN + n-hexane (2:1)					
22.	CH_3NO_2 + n-hexane (2:1)					
23.	THF + n-hexane (2:1)					
24.	1,2-Dioxane + n-hexane (2:1)					

Melt Crystallization:

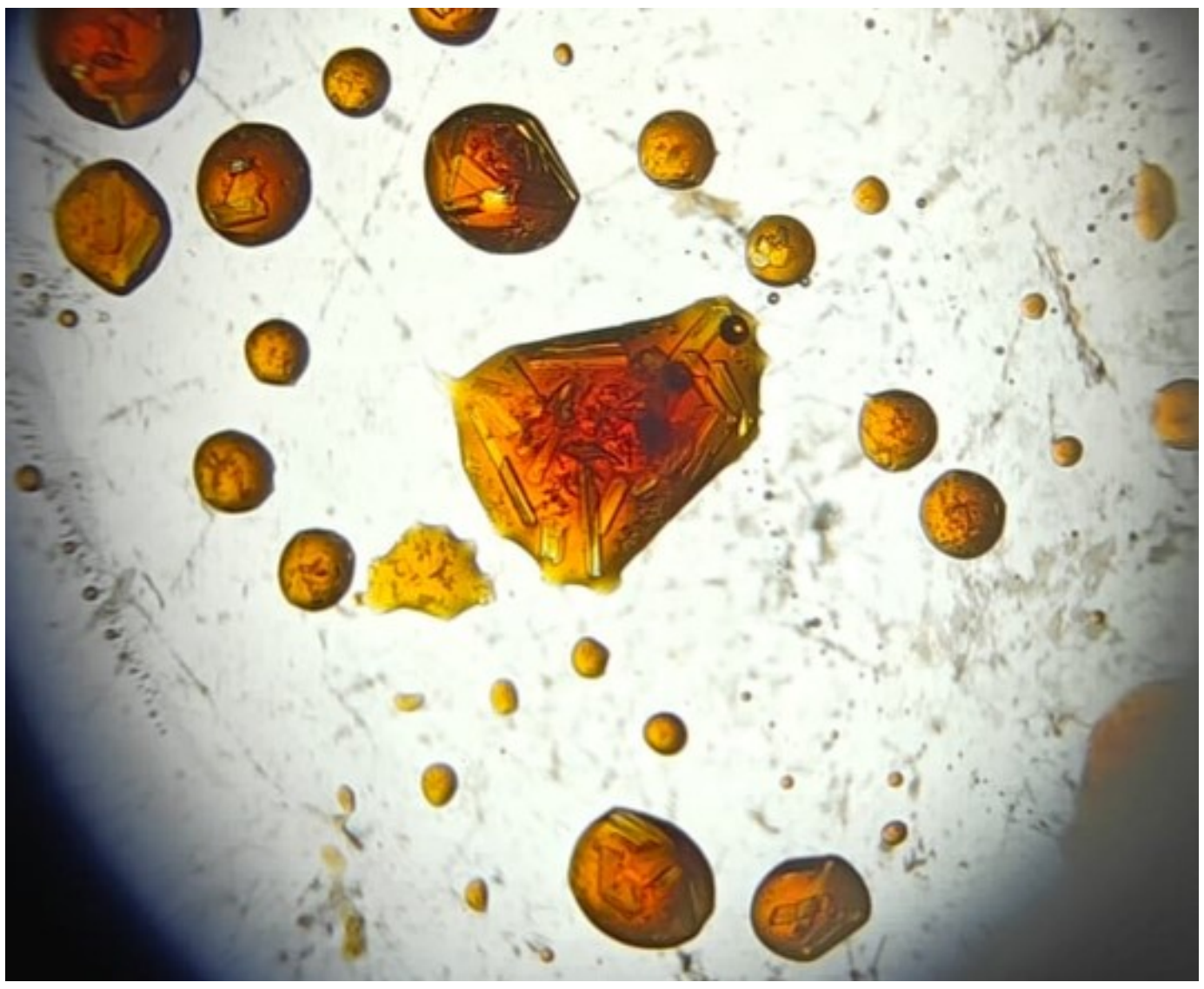

Figure S7. Optical image of the crystals formed from melt crystallization.

Table S2. Pairwise RMSD and maximum atomic displacement (Max. D) values obtained from molecular overlay analysis in between the Forms **I** – **V**.

**Polymorphs Pairs**	**RMSD**	**Max. D**
**I** vs **II**	1.0202	2.3228
**I** vs **III**	0.2621	0.6273
**I** vs **IV**	1.1445	2.7894
**I** vs **V**	0.7446	1.7318
**II** vs **III**	1.1081	2.6923
**II** vs **IV**	0.5168	1.2300
**II** vs **V**	1.1515	2.4434
**III** vs **IV**	1.1975	3.0370
**III** vs **V**	0.7733	1.7607
**IV** vs **V**	1.2865	2.8034

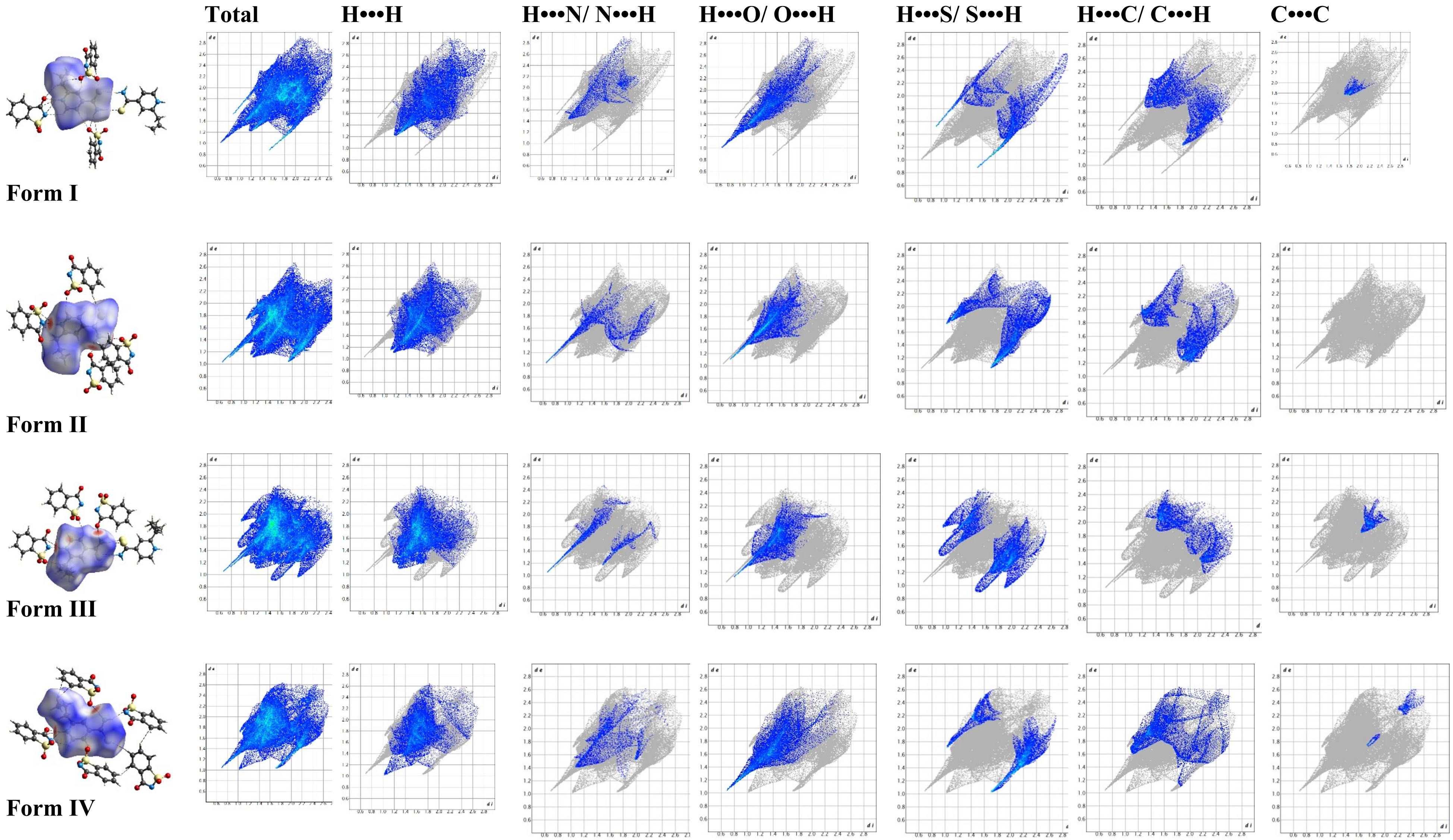
Total
H•••H
H•••N/ N•••H
H•••O/ O•••H
H•••S/ S•••H
H•••C/ C•••H
C•••C
Form I
Form II
Form III
Form IV

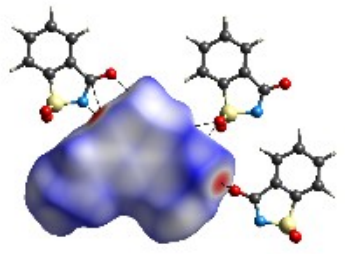

Form V

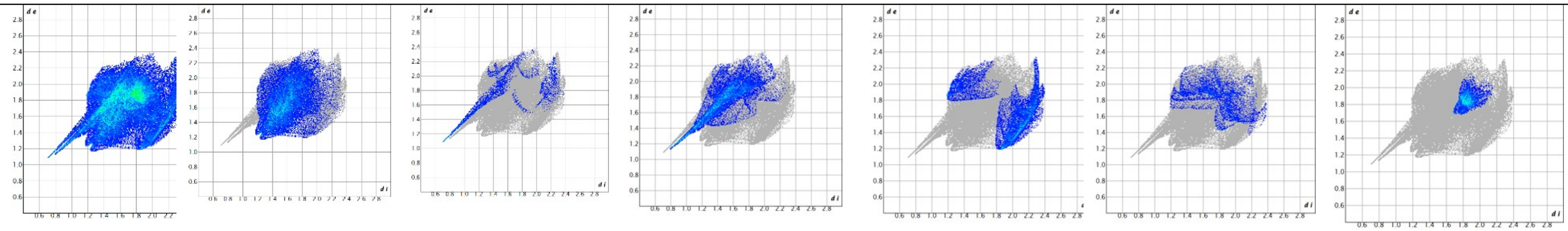

Figure S8. Hirshfeld surface and corresponding fingerprint plot of all Forms**.**

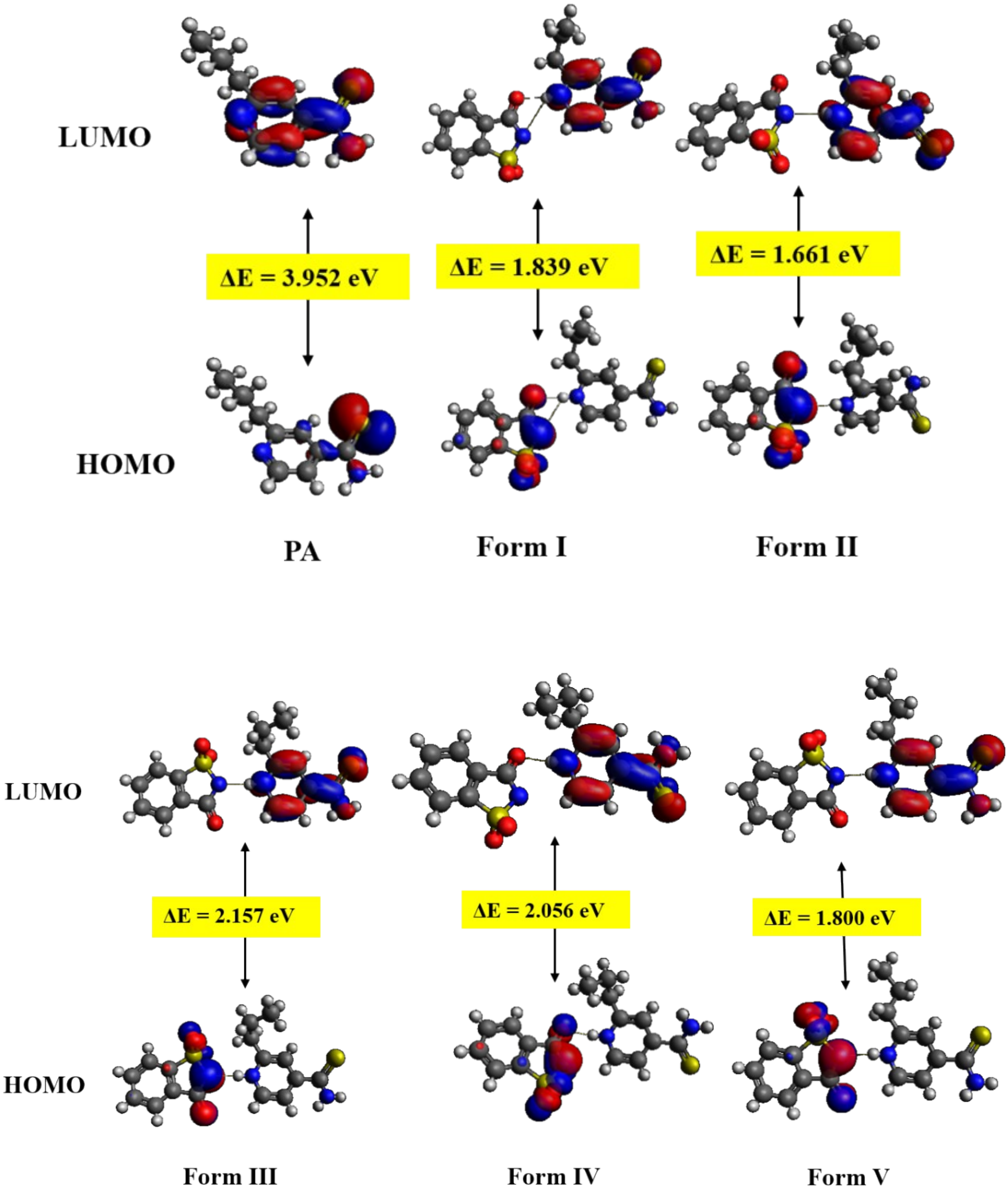

Figure S9. Molecular orbitals of native **PA** and Forms **I** - **V**.

Thermodynamics Explanation about the transformation Form III → Form V transformation:

	Form **III**	Form **V**
Transition Temperature (T_{tr})	99.79 °C (372.79 K)	
Melting Temperature (T_m)	115.60 °C (388.60 K)	123.087 °C (396.087 K)
Enthalpy of melting (ΔH_m)	17.20 J/g<br>= 17.20 × 363.44 J/mol<br>= 6251.17 J/mol	20.91 J/g<br>= 20.91 × 363.44 J/mol<br>= 7599.53 J/mol
Enthalpy of transition, (ΔH_{tr})	(20.91 – 17.20) = 3.71 J/g<br>= 3.71 × 363.44 J/mol<br>= 1348.3624 J/mol	
	Molar mass = 363.44 g/mol (for both)	

Melting entropy: $\Delta S_m = \Delta H_m/T_m$

(ΔS_m) for Form **III** = 6251.168/388.60 = 16.0863 J $mol^{-1}K^{-1}$;

(ΔS_m) for Form **V** = 7599.5304/396.087 = 19.1865 J $mol^{-1}K^{-1}$

Transition entropy $\Delta S_{tr} = \Delta H_{tr}/T_{tr}$: 1348.3624/372.79 = 3.6169 J $mol^{-1}K^{-1}$

Gibbs free energy; $G = H - TS$

Let's consider, Gibbs free energy Form **III** at 0 K assumed zero

$G(T)_{III} = H_0\,(III) - TS\,(III)$

For Form V (including transition enthalpy and entropy relative to Form III):

$G_V\,(T) = G\,(III)\,(T) + \Delta H_{tr} - T\Delta S_{tr}$

Assume H_0, Form III = 0 (reference).

$\mathbf{S_{III}}$ and $\mathbf{S_V}$ are approximated by melting entropies.

**Temperature (K)**	**G_{III} (J/mol)**	**G_V (J/mol)**
200	−3217.26	-2592.28
300	−4825.89	-4562.60
372.79 (trans.)	−5996.81	-5996.79

388.60 (melting Form III)	−6251.1361	−6251.1178
396.087 (melting Form V)	NA	−84.25

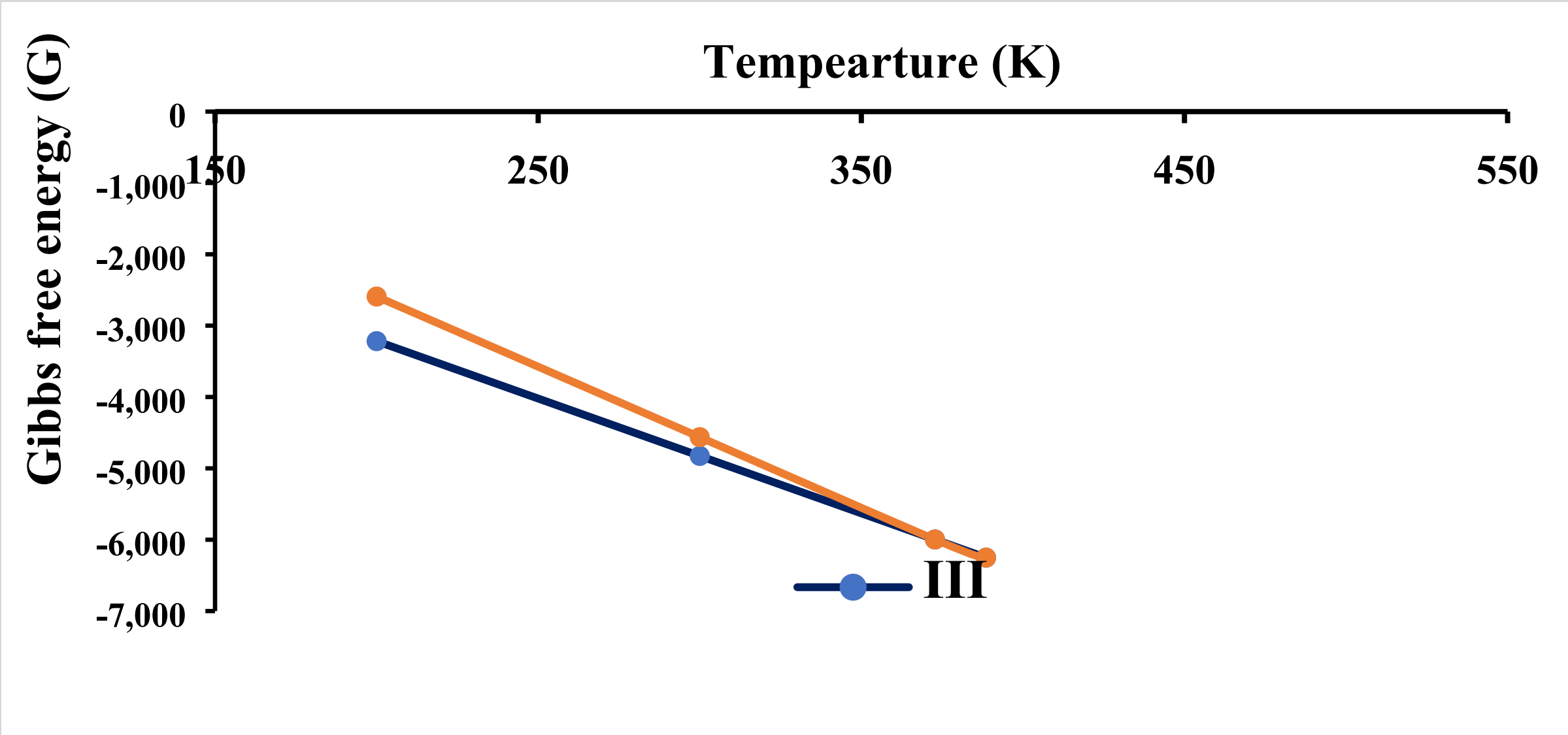

Figure S10. Gibbs free energy (G) versus temperature for Forms **III** and **V**.

From the above graph, the Gibbs free energy curves of Form **III** and Form **V** are very close and appear to intersect around 350 K. This is consistent with an enantiotropic relationship.
